# A Review of Botany, Phytochemistry, and Biological Activities of Eight *Salvia* Species Widespread in Kazakhstan

**DOI:** 10.3390/molecules30051142

**Published:** 2025-03-03

**Authors:** Yana Levaya, Gayane Atazhanova, Vika Gabe, Karakoz Badekova

**Affiliations:** 1 School of Pharmacy, Karaganda Medical University, Gogol Street, 40, Karaganda 100017, Kazakhstan; g-atazhanova@mail.ru (G.A.); karlito42@mail.ru (K.B.); 2 Department of Physiology, Biochemistry, Microbiology and Laboratory Medicine, Institute of Biomedical Sciences, Faculty of Medicine, Vilnius University, 03101 Vilnius, Lithuania; vika.gabe@mf.vu.lt

**Keywords:** botany, phytochemistry, biological activities, traditional medicine, medicinal plants, bioactive compounds

## Abstract

The aim of this review is to provide a comprehensive overview of the botany, phytochemistry, and biological activities of eight *Salvia* species, namely *Salvia aethiopis* L., *S*. *sclarea* L., *S*. *dumetorum* Andrz. ex Besser, *S*. *deserta* Schang., *S*. *trautvetteri* Rgl., *S*. *macrosiphon* Boiss., *S*. *virgata* Jacq., and *S*. *verticillata* L., which are widespread in Kazakhstan. The genus *Salvia* is renowned for its diverse medicinal properties, and these species are no exception, contributing to the rich natural pharmacopoeia of the region. The botanical characteristics of these species, including their morphological features, distribution, and ecological adaptations, are discussed. The present review also explores the phytochemical composition of these plants, focusing on bioactive compounds such as terpenoids, flavonoids, alkaloids, and phenolic acids, which are responsible for their medicinal potential. Biological activities including antimicrobial, antioxidant, anti-inflammatory, antidiabetic, and neuroprotective effects are evaluated based on available in vitro and in vivo studies. In addition, the review highlights the traditional uses of these species in local medicine and suggests avenues for future research to further elucidate their pharmacological potential. This synthesis provides valuable insights into the medicinal importance of these *Salvia* species in Kazakhstan and supports their continued exploration for therapeutic applications.

## 1. Introduction

Currently, the genus *Salvia* L. (family Lamiaceae) comprises 1024 accepted species according to the taxonomic list by POWO [1] and is one of the largest genera of perennial and shrubby plants within the family [2]. In the flora of Kazakhstan [3], eight species of this genus are listed: *Salvia aethiopis* L., *S. sclarea* L., *S. dumetorum* Andrz. ex Besser, *S. deserta* Schang., *S. trautvetteri* Rgl., *S. macrosiphon* Boiss., *S. virgata* Jacq., and *S. verticillata* L.

Sage is an aromatic medicinal plant that was referred to as the “holy herb” in ancient times and used as a tonic, antiseptic, antipyretic, and astringent. One of the most well-known representatives of the *Salvia* genus is *Salvia officinalis* L. The name *salvia* translates from Latin as “unharmed, whole”. It is native to Italy and Southeastern Europe. Currently, the plant is actively cultivated, including in Russia [4]. The leaves of this species are included in the official pharmacopoeias of Kazakhstan [5], Europe [6], Russia [7], Ukraine [8], Britain [9], and the USA [10]. The essential oil (EO) and extracts of *S. officinalis* are widely used in traditional medicine, culinary arts, food industry, cosmetics, and perfumery.

The chemical composition of *S. officinalis* is characterized by a variety of biologically active substances (BASs). The medicinal properties of the plant are primarily associated with its EO, which consists predominantly of thujone, as well as camphor, diterpenes, triterpenes, tannins, estrogens, and phenolic acids (such as chlorogenic, rosmarinic, and caffeic acids, among others) [11]. Many of these substances are ingredients in anti-inflammatory, analgesic, and antiseptic drugs, and expectorants. Additionally, sage is rich in flavonoids (rutin, quercetin, and hyperoside)—antioxidants with antimicrobial properties found in heart medicines [12]. The leaves contain alkaloids, flavonoids, tannins, linoleic, oleanolic, and ursolic acids, essential for lipid metabolism [13]. Due to its diverse chemical composition, sage has broad potential applications such as in anti-inflammatory, antiseptic, immunomodulating, hypoglycemic, antidiabetic, antioxidant, antitumor, hepatoprotective, and wound healing agents [14].

Thus, *Salvia* is an important medicinal plant, like many others, containing diverse classes of BASs and exhibiting pharmacological activity. The aim of this review is to gather and systematize existing data on the chemical composition of eight species of sage growing in Kazakhstan. This is due to the limited and unsystematized amount of information and publications compared to the more common species of the genus *Salvia.* Additionally, given the sufficient availability and demand for sage plants in medicine and pharmacy, developing new medicinal products based on new data collection is relevant. Some sage species have not been studied for the content of BASs at all, or only certain classes of compounds have been studied. Due to the wide range of health benefits associated with *Salvia*, this review will help to form a current understanding of the situation regarding individual sage species and assess their effectiveness and safety for further research and development in the medical and pharmaceutical fields.

## 2. Botany and Distribution of the Genus *Salvia* in Kazakhstan

**Figure 1:**
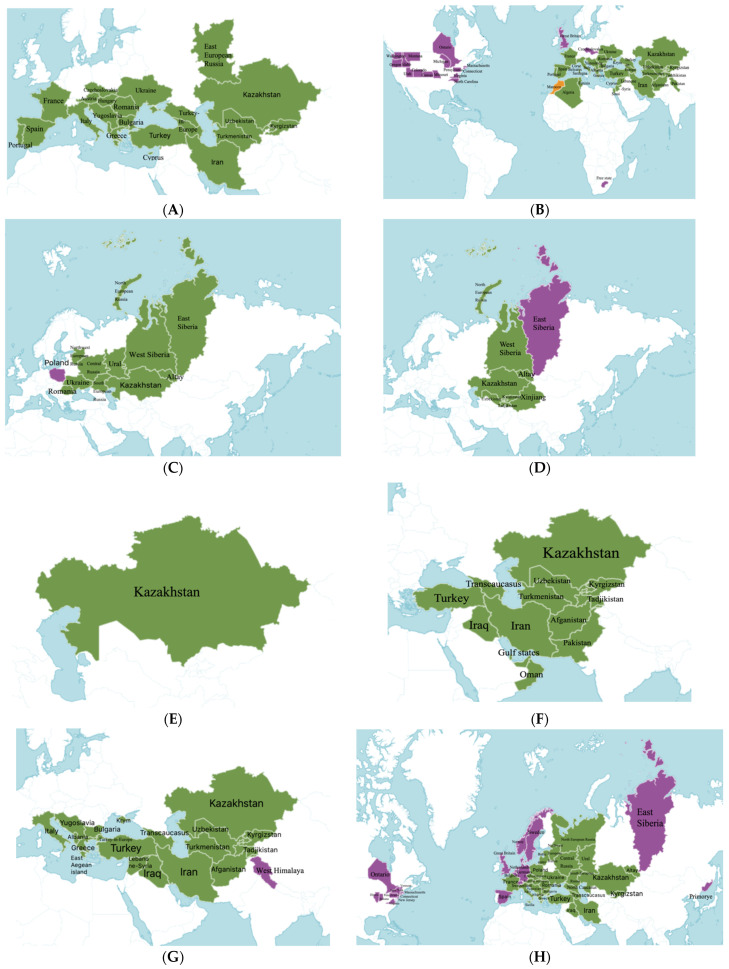
Distribution of wild and cultivated species of *Salvia* genus worldwide [1]: (**A**) *S. aethiopis*; (**B**) *S. sclarea*; (**C**) *S. dumetorum*; (**D**) *S. deserta*; (**E**) *S. trautvetteri*; (**F**) *S. macrosiphon*; (**G**) *S. virgata*; and (**H**) *S. verticillata*. green—native and violet—introduced.

The life forms of *Salvia* spp. include perennial herbaceous shrubs and subshrubs, with biennial species less common than annual ones [3]. They are characterized by erect or ascending stems, which are often four-angled and variably pubescent. The leaves are typically entire, sometimes toothed or deeply lobed, and rarely pinnately dissected. Stem leaves vary in shape and size compared to basal leaves. The flowers of these plants are brightly colored and often shiny, sessile, or borne on short pedicels, arranged in dense verticillasters, spike-like, racemose, or paniculate inflorescences. Bracts and leaves are small and persistent. The calyx is usually tubular or bell-shaped, sometimes funnel-shaped, with two lips, covered by a membrane and enlarging as the fruits mature. The upper lip is often truncate or atrophied, sometimes spreading and irregularly three-toothed, while the lower lip always has two lobes. The corolla also typically has two lips. The corolla tube is cylindrical, widening upwards and may be straight or curved. The upper lip of the corolla is usually straight, occasionally concave or arched. The lower lip is spreading, with three lobes, the central lobe being large and deeply notched or lobed. There are usually two fertile stamens on reduced filaments, and two stamens may be reduced or absent. The fruit is always double, dry, splitting into four nutlets. Species of this genus are distributed worldwide, including Kazakhstan, Europe, Asia, Russia, and the USA, with introduced species noted in Russia and partially in the USA and European countries (Figure 1) [1].

Plants of the genus *Salvia* flower for an extended period and profusely, with flowering times varying by species and cultivar. All species of this genus are aromatic plants, many traditionally used as medicinal herbs, though many remain to be thoroughly studied.

Based on international representations, there are eight species of this genus growing in Kazakhstan, the morphological characteristics of these species are presented in Table 1.

Thus, the main differences between the species are in the structure of the leaf shape, the size of the stems and inflorescences, the structure of the calyx, and the color of the corolla of the flower. The habitat of all species is mainly confined to the steppe territories, since these plants are very demanding on light, so they grow better in an open, sunny area on the south side. On the territory of Kazakhstan, two species have a wide range, beyond its borders covering Western Siberia, Altai and Central Asia. One species is an endemic, whose range includes only the city of Karatau in Southern Kazakhstan.

## 3. Extraction Methods

To extract BASs from plants of the *Salvia* genus, a wide range of extraction methods are utilized. Among the most common methods are conventional techniques such as maceration, percolation, Soxhlet extraction, and hydrodistillation. However, these methods have several drawbacks, primarily related to increased extraction time, the requirement for expensive and bulky equipment, and the potential loss of BASs due to hydrolysis, oxidation, and ionization processes during extraction [15]. In contrast, supercritical carbon dioxide extraction, the deep eutectic solvent extraction method, pressurized liquid extraction, enzyme-assisted extraction, ultrasonic extraction, and microwave-assisted extraction (MAE) are less common and less studied methods for obtaining extracts from sage.

Based on a literature review of eight species of sage growing in Kazakhstan, it is noted that studies on extracts obtained by modern extraction methods are quite limited. Comparative data on extraction methods for different sage species, plant parts used, and solvents employed are presented in Table 2. It was found that maceration is the most common method used for obtaining extracts from *S. virgata*, *S. macrosiphon*, and *S. aethiopis*, whereas ultrasonic extraction is preferred for *S. dumetorum* and *S. sclarea*.

One of the interesting solutions proposed by scientists from Ukraine [30] for extracting BASs from plant material involved a study comparing combined extraction methods ultrasound + maceration and percolation + maceration with maceration. The authors investigated total phenolic content (TPC) and total flavonoid content (TFC) in extracts from the aerial parts of *S. sclarea*. The extract yield for ultrasound + maceration was found to be the highest among the studied extracts, exceeding percolation + maceration by 8% when using 70% ethanol. However, despite its high yield, the extract obtained by maceration exhibited the highest TPC and TFC contents, measuring 1045.9 mg eq-GA/g and 2103.2 mg eq-RU/g, respectively.

Nowadays, SFE-CO_2_ is one of the most popular and effective methods for obtaining extracts from plant material. Sulniute et al. [25], for the extraction of *S. sclarea*, *S. dumetorum*, and *S. verticillata* herbs collected in Lithuania during the flowering phase, used the SFE-CO_2_ method followed by processing the CO_2_ extract with 96% ethanol and water. The extracts were identified using ultra-high performance liquid chromatography with quadrupole time-of-flight (UPLC–Q/TOF), revealing several phenolic acids and flavonoids. Ethanol extracts showed a higher content of phenolic acids compared to flavonoids. In contrast, water extracts exhibited the opposite trend, while carbon dioxide extracts contained minimal to no phenolic compounds, behaving more like impurities.

The authors in [28] proposed a green extraction method for *S. sclarea* using a natural deep eutectic solvent (NADES) composed of citric acid and glucose. During this study, the content of rosmarinic acid and luteolin in three extracts from the aerial parts of *S. sclarea* was determined. Methanol was used for maceration, while ultrasound and NADES were employed for percolation. The results indicated that the highest content of rosmarinic acid, 191.1 µg/g, was found in the methanolic extract obtained using ultrasound.

Another study by Levaya et al. [43] investigated antibacterial activity of ethanolic extracts from *S. dumetorum* leaves grown in Kazakhstan, using MAE. This method is widely applied for obtaining extracts from *S. officinalis* [44,45], as it has been repeatedly demonstrated to be a more efficient method for extracting polyphenols from plant material compared to other methods [46]. MAE was also utilized for obtaining hydrosols and EOs from *S. aethiopis* and *S. sclarea* leaves [47].

Also, several studies were found using ultrasound extraction to obtain secondary metabolites from different *Salvia* species. Extracts of *S. sclarea* [28,31], *S. dumetorum* [24,33], *S. verticillata* [33,34,36], and *S. aethiopis* [33,41] were obtained from the aerial part, mostly from leaves, using methanol and methanol solutions. Comparing *S. sclarea* aerial part methanolic extracts from Turkey [28] and from Serbia [31], it was found that *S. sclarea* extract from Turkey had rosmarinic acid content of 191.1 mg/g and from Serbia had rosmarinic acid content of 177.77 mg/g. Thus, the qualitative and quantitative composition of the extract depends directly not only on the plant organ being extracted, its geographical origin, and the solvent used, but also on the extraction method and conditions.

Therefore, based on the literature review conducted, it can be concluded that there are numerous extraction methods for the genus *Salvia*. Each of the listed methods has its own advantages and disadvantages, and the choice in each case depends on the extraction objectives. Modern extraction methods are widely used globally for obtaining plant extracts [48]; however, these methods are rarely utilized for extracts from the *Salvia* species and are discussed in this article. Thus, this direction could be promising in terms of generating new data on the qualitative and quantitative composition of these plants.

## 4. Phenolic Compounds and Terpenoids

The genus *Salvia* is now known to be an overproducer of phenolpropanoids and rosmarinic acid. Of particular interest from a scientific point of view are phenolic acids and flavonoids, which have been found in the raw materials of some *Salvia* species [49]. Phenolic compounds are an extensive class of compounds of various groups, presented as follows: hydroxybenzoic acids, hydroxycinnamic acids, phenolic acids, flavones, flavonoids, phenolic diterpenes, and coumarins (Table 3).

The authors [16] conducted a study of the TPC and TFC of methanolic extracts obtained by maceration from eight sage species growing in Iran. It was found that the highest content of phenolic compounds in *S. macrosiphon*, *S. virgata*, and *S. sclarea* is found in leaves (TPC 14.7–35.6 mg eq-GAE/g and TFC 51.9–83.9 mg eq-QE/g); however, the flowers of *S. virgata* also contained high levels of TPC and TFC, 36.7 mg eq-GAE/g and 100.4 mg eq-QE/g, respectively.

This study [29] aimed to evaluate the TPC, TFC, and content of phenolic acids of *S. sclarea* ethanol extracts during the vegetation period. TPC content was 9.39–91.02 mg eq-GAE/g^−1^ and the minimum was found at the flowering stage. The total content of phenolic acids was 0.13–36.01 mg eq-CAE/g^−1^ and the minimum was determined at the budding stage. The highest content of TFC, 25.91 to 53.82 mg eq-QE/g^−1^, was found in leaf extracts at budding and fruitage, in inflorescences extracts at the flowering stage. Another study [30] from Ukraine investigated the yield of TPC from *S. sclarea* herb and determined that TPC was the highest in the extracts prepared by heating at a temperature of 36–46 °C prepared with a solvent-to-herb ratio (10:1) and particle size 2–5 mm and quantified as 1045.9 mg eq-GA/L and 2103.12 mg eq-RU/L.

The authors in [24] reported that caffeic (**1**) and rosmarinic (**2**) acids are the most abundant phenoloids from the ethanolic extract of *S. sclarea* and *S. dumetorum* leaves. *S. dumetorum* extract has higher amounts of compounds (**1**, **2**) and TPC (2.09, 0.08 and 6.54%) than *S. sclarea* extract (0.87, 0.05 and 3.07%).

**Figure 2:**
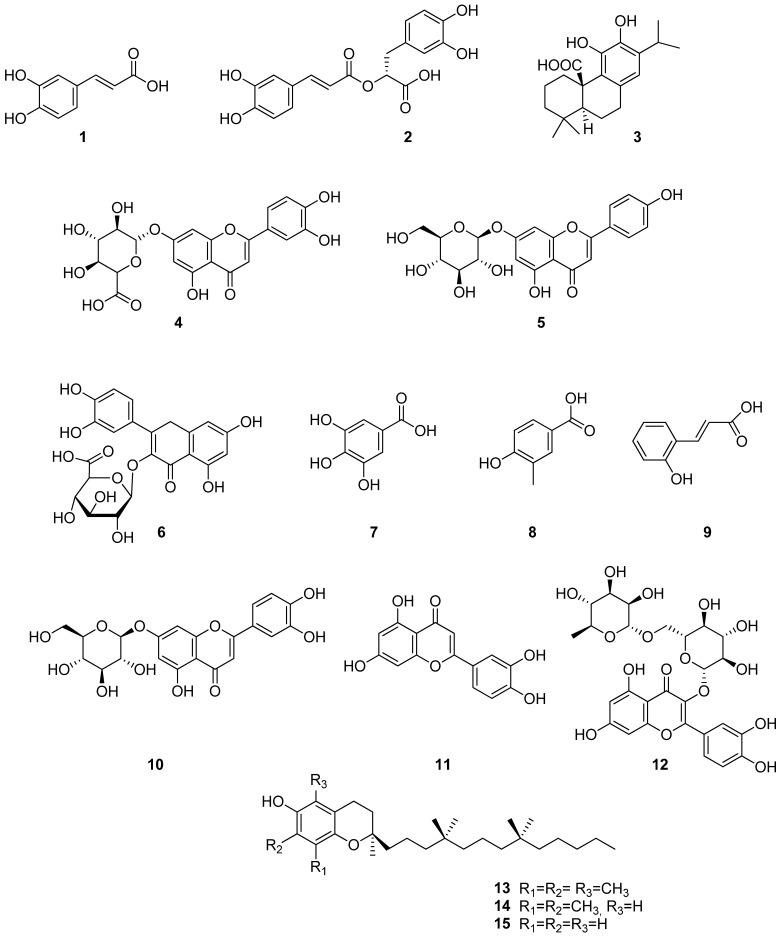
Chemical structures of phenolic compounds found in *Salvia* species. Part 1.

The identification of extracts from *S. sclarea*, *S. dumetorum*, and *S. verticillata*, obtained using SFE-CO_2_ followed by treatment with CO_2_ extract, 96% ethanol, and water, was conducted using UPLC–Q/TOF, during which several phenolic acids and flavonoids were detected [25]. It was found that ethanol extracts exhibited higher levels of phenolic acids compared to flavonoids (Figure 2).

In aqueous extracts, an inverse relationship was observed, while in carbon dioxide extracts, the content of phenolic compounds was at the level of impurities or was absent altogether. Thus, caffeic (**1**), rosmarinic (**2**), and carnosic (**3)** acids were found in ethanol extracts, while luteolin-7-glucuronide (**4**), apigenin-7-glucuronide (**5**), and quercetin-3-glucuronide (**6**) were found in aqueous extracts. The highest concentration of compounds (**1**–**3**) was determined in the *S. verticillata* ethanol extract, whereas the highest concentration of compounds (**4**–**6**) was found in the *S. sclarea* ethanol extract.

In another study [17], *S. virgata* ethyl acetate, methanol, 50% methanol, and water extracts were identified using high-performance liquid chromatography coupled with photo diode array (HPLC–PDA) analysis and showed the presence of caffeic (**1**), rosmarinic (**2**), gallic (**7**), *p*-OH-benzoic (**8**), and *o*-coumaric (**9**) acids, cynaroside (**10**), and luteolin (**11**). All extracts were found to be rich in rosmarinic acid with 4.48–59.75 mg/g, except for the hexane extract. The major phenolic compounds in *S. virgata* methanolic extract were found to be rutin (**12**) and rosmarinic acid (**2**) [18]. A recent study indicated that the TPC of *S. virgata* ethanolic extract is higher than TFC (283.35 ± 10.4 mg eq-GAE/g and 13.37 ± 1.6 mg eq-QE/g, respectively) [20]. The authors in [21] studied the TPC regarding the catechin equivalent for *S. virgata* from Iran at 7.5 mg eq-CE/g dry weight and *S. macrosiphon* at 7.1 mg eq-CE/g dry weight of methanolic extracts. The results of this study revealed that phenolic compounds play a key role in the biological activity of extracts.

The authors in [16] conducted a study of the TPC and TFC of methanolic extracts obtained by maceration from eight sage species growing in Iran. It was determined that the highest content of phenolic compounds was found in the leaves of *S. macrosiphon*, *S. virgata*, and *S. sclarea* (TPC 14.7–35.6 mg eq-GAE/g and TFC 51.9–83.9 mg eq-QE/g); however, the flowers of *S. virgata* also contained high levels of TPC and TFC 36.7 mg eq-GAE/g and 100.4 mg eq-QE/g, respectively.

Sulniute et al. [25] investigated the yield of α-tocopherol (**13**), γ-tocopherol (**14**), and δ-tocopherol (**15**) isolated from *S. dumetorum*, *S. sclarea*, and *S. verticillata* using SFE-CO_2_. The concentration of γ-tocopherol was remarkably low, while δ-tocopherol was detected only in *S. verticillata* and was not detected in *S. sclarea* and *S. dumetorum* extracts. It should be noted that α-tocopherol is the most biologically active isomer and the highest amount was 8364 µg/g dry weight of *S. verticillata* extract.

**Figure 3:**
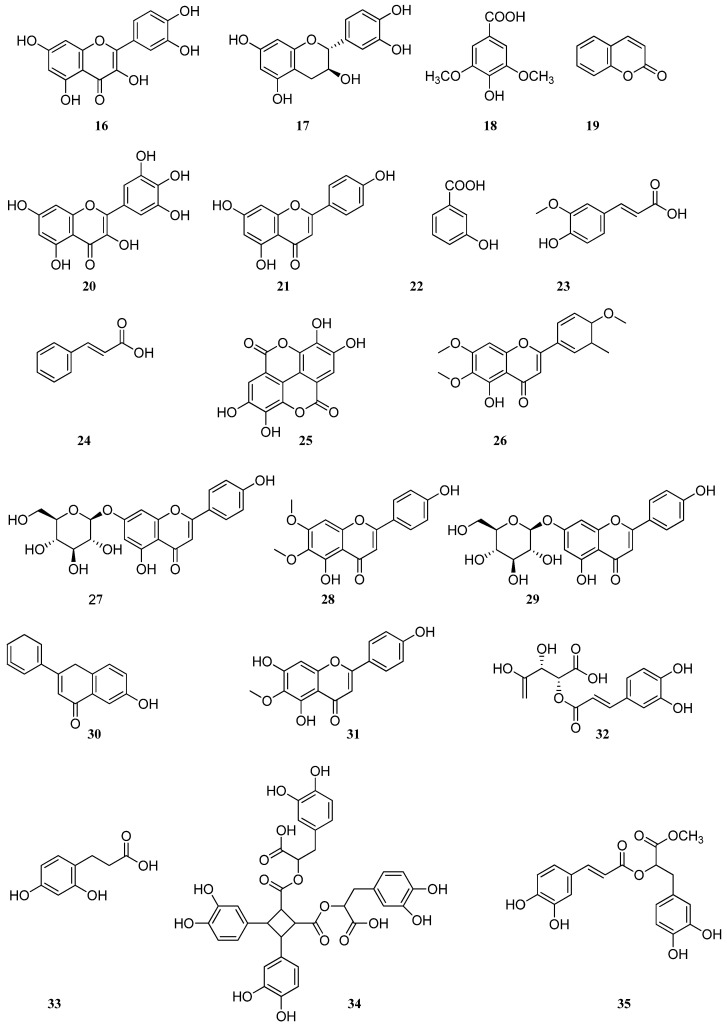
Chemical structures of phenolic compounds found in *Salvia* species. Part 2.

High-performance liquid chromatography with diode-array detection (HPLC–DAD) was used to determine the phenolic composition of methanolic, ultrasonic, and green extracts of *S. sclarea* [28]. The results showed that rosmarinic acid (**2**) and quercetin (**16**) were in high quantities in all extracts. The highest concentrations of compounds (**2**) and (**16**) were found in ultrasonic extract, and were found to be 191.1 ± 0.27 and 25.01 ± 0.30 μg/g, respectively, followed by methanolic extract at 179.5 ± 0.45 and 24.48 ± 0.22 μg/g, respectively, followed by green extract at 90.31 ± 0.36 and 4.41 ± 0.25 μg/g, respectively. In methanolic and ultrasonic extracts of *S. sclarea* from Turkey other phenolic compounds were also found including the following: caffeic acid (**1**), gallic acid (**7**), luteolin (**11**)**,** rutin (**12**), catechin (**17**), syringic acid (**18**), coumarin (**19**), myricetin (**20**), apigenin (**21**), 3-hydroxy benzoic acid (**22**), ferulic acid (**23**), and *trans*-cinnamic acid (**24**) (Figure 3). However, compounds (**22**–**24**) were not found in green extract, and the ellagic acid (**25**) (3.40 ± 0.11 μg/g) was present only in green extract.

Research conducted by scientists from Serbia on the aerial parts of *S. sclarea* confirms previous findings that rosmarinic acid (**2**) is the dominant compound in extracts obtained by maceration and ultrasound extraction [31]. The chemical composition was determined using HPLC, revealing caffeic acid (**1**) and rosmarinic acid (**2**) in amounts ranging from 0.63 to 0.97 and from 171.99 to 197.48 μg/mg, respectively. It was established that for the selective extraction of phenolic acids and flavonoids, a methanol–water solution is required, whereas absolute methanol is used to increase the amount of aglycones in the extract. The *S. sclarea* extracts from Serbia contain aglycones such as luteolin (**11**), apigenin (**21**), and salvigenin (**26**), as well as glycosides including cynaroside (**10**) and cosmosiin (**27**).

The authors in [33] investigated the content of individual phenolic compounds in *S. dumetorum*, *S. aethiopis*, and *S. verticillata* growing in Ukraine using HPLC–DAD. The chemical composition of *S. dumetorum*, *S. aethiopis*, and *S. verticillata* was characterized by the presence of caffeic (**1**) and rosmarinic (**2**) acids, cynaroside (**10**), rutin (**12**), and cirsimaritin (**28**). *S. dumetorum* contained kaempferol-3-*O*-glucoside (**29**), *S. aethiopis* and *S. verticillata* contained cosmosiin (**27**), *S. verticillata* contained 6-hydroxyluteolin-5-glucoside (**30**), *S. aethiopis* contained apigenin (**21**), and *S. aethiopis* and *S. dumetorum* contained hispidulin (**31**). In addition, *S. verticillata* leaves had the richest amount of rosmarinic acid (**2**) quantified as 12,310 mg/kg. The claim supported by other research [34] determined that rosmarinic acid (**2**) was the most abundant phenolic compound of *S. verticillata*.

**Figure 4:**
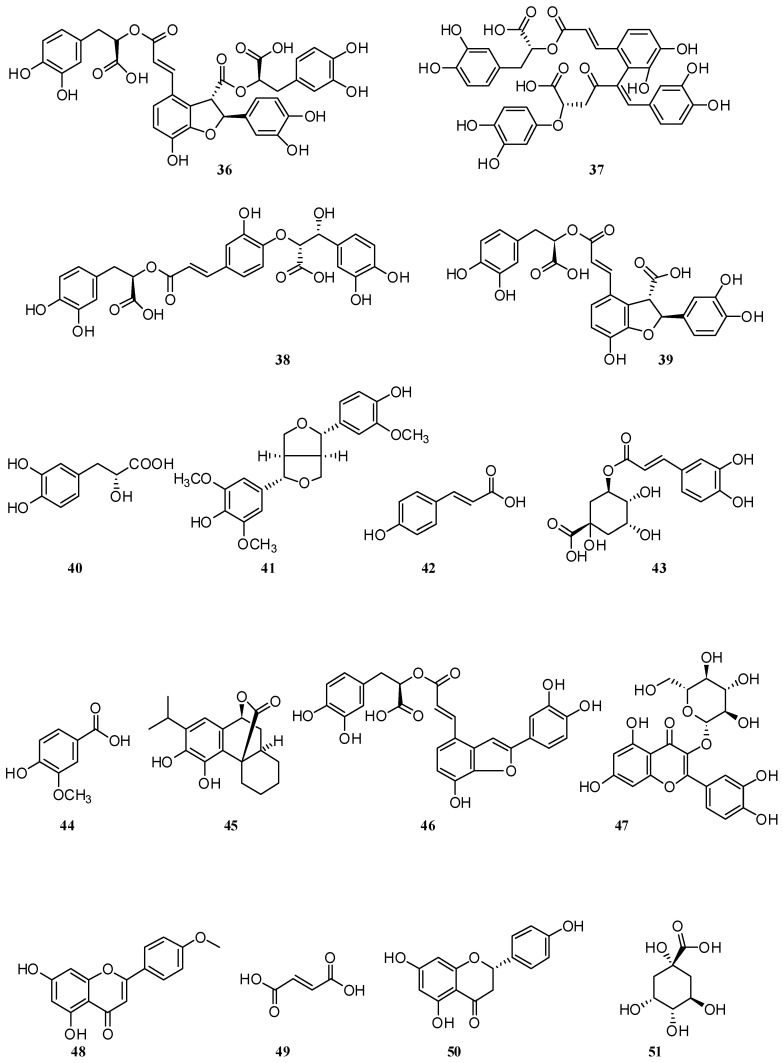
Chemical structures of phenolic compounds found in *Salvia* species. Part 3.

Twenty-eight compounds were detected in *S. verticillata* leaf hydromethanolic extracts from Greece using high-performance liquid chromatography coupled with diode array detection and electrospray ionization mass spectrometry (HPLC–DAD–ESI–MS) [36], among them the following thirteen phenolic acids: rosmarinic acid (**2**), caftaric acid (**32**), dimer-β-(3,4-dihydroxyphenyl) lactic acid (**33**), sagerinic acid (**34**), methyl-rosmarinic acid (**35**), salvianolic acid B (**36**), salvianolic acid E (**37**), salvianolic acid K (**38**), lithospermic acid (**39**), danshensu (**40**), and medioresinol (**41**) (Figure 4), and five flavones: glucuronides of luteolin (**4**), apigenin (**21**), salvigenin (**26**), cirsimaritin (**28**), and hispidulin (**31**).

Another study [37] indicated the presence of some new compounds in *S. verticillata* leaves from Turkey which have not been previously described for *S. verticillata*, such as gallic acid (**7**), o-coumaric acid (**9**)**,** ferulic acid (**23**), *trans*-cinnamic acid (**24**)**,** p-coumaric acid (**42**), chlorogenic acid (**43**), and vanillic acid (**44**). A total of 29 phenolic compounds were detected by UHPLC-MS4 orbitrap metabolic fingerprinting data of *S. verticillata* extract; 21 constituents were phenolic acids and their derivatives and 8 constituents were flavonoids and their derivatives [38]. In this study, two hexuronyl derivatives, luteolin 7-glucuronide (**4**) and apigenin 7-glucuronide (**5**), and diterpenes, such as carnosol (**45**) and salvianolic acid C (**46**), were identified.

**Figure 5:**
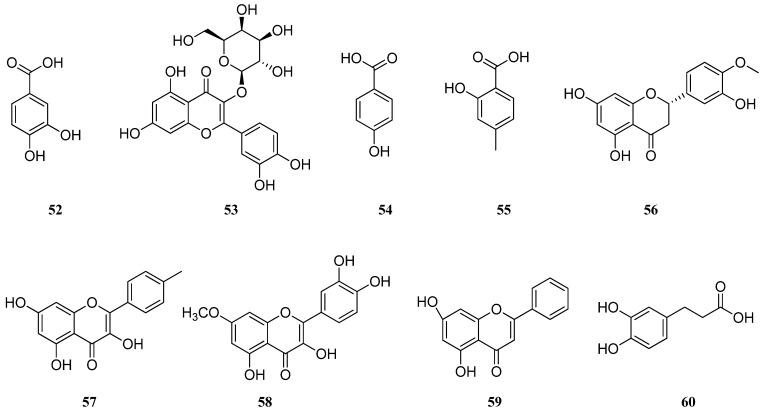
Chemical structures of phenolic compounds found in *Salvia* species. Part 4.

When comparing studies on *S. aethiopis* from European and Asian parts of the world, differences in the component composition of the extracts were found. The HPLC method was used for the quantification of phenolic compounds of *S. aethiopis* and *S. sclarea* extracts [39]. It was revealed that *S. aethiopis* contained a high amount of isoquercetin (**47**) (20.52 ± 1.7 mg/g), caffeic acid (**1**) (18.28 ± 2.3 mg/g), acecetin (**48**) (11.34 ± 0.8 mg/g), and fumaric acid (**49**) (9.86 ± 0.9 mg/g). On the other hand, naringenin (**50**) (20.76 ± 3.8 mg/g), caffeic acid (**1**) (17.13 ± 0.3 mg/g), luteolin (**11**) (10.27 ± 0.4 mg/g), and isoquercetin (**47**) (9.05 ± 0.8 mg/g) were found to be high in *S. aethiopis* extract. The authors identified 17 compounds from the *S. aethiopis* extract using liquid chromatography with tandem mass spectrometry (LC–MS/MS), with phenolic compounds being the dominant components [40]. Among them were luteolin (**11**), apigenin (**21**), *p*-coumaric acid (**42**), chlorogenic acid (**43**), naringenin (**50**), quinic acid (**51**), protocatechuic acid (**52**), hyperoside (**53**), 4-hydroxybenzoic acid (**54**), salicylic acid (**55**), hesperetin (**56**), kaempferol (**57**), rhamnetin (**58**), and chrystin (**59**) (Figure 5).

**Figure 6:**
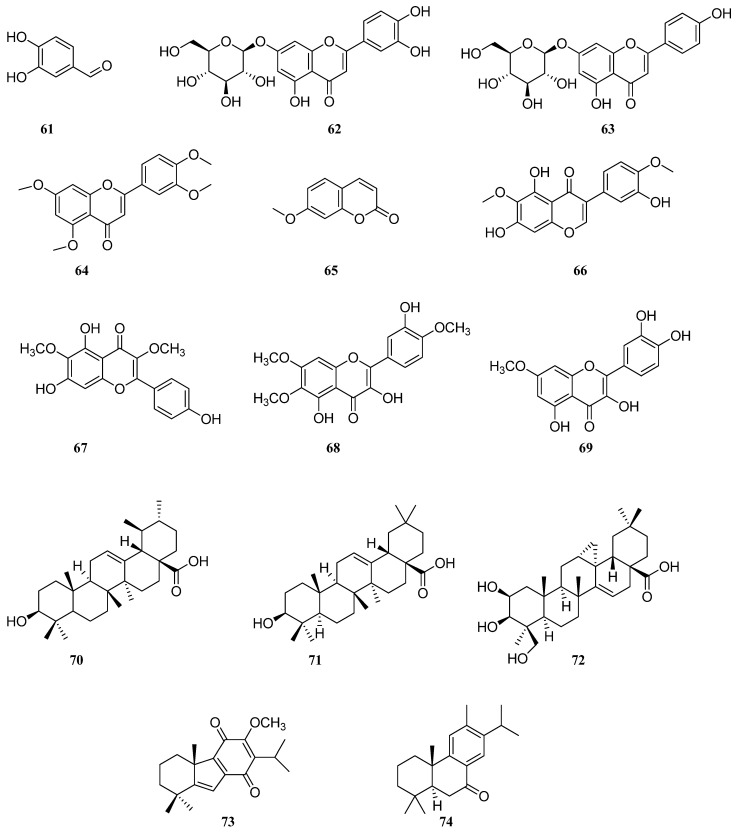
Chemical structures of phenolic compounds found in *Salvia* species. Part 5.

There was a publication regarding *S. deserta* extracts. The authors investigated the LC–DAD–QTOF-MS of *S. deserta* and *S. sclarea* from Kazakhstan [32]. Results showed the presence of caffeic acid (**1**), rosmarinic acid (**2**), luteolin 7-glucuronide (**4**), apigenin 7-glucuronide (**5**), luteolin (**11**), apigenin (**21**), salvigenin (**26**), cirsimaritin (**28**), salvianolic acid B (3**6**), salvianolic acid K (**38**), danshensu (**40**), vanillic acid (**44**), dihydrocaffeic acid (**60**), protocatechuicaldehyde (**61**), luteolin 7-*O*-glucoside (**62**), apigenin 7-*O*-glucoside (**63**), tetramethoxeflavone (**64**), methoxycoumarin (**65**), jaceosidin (**66**), viscosine (**67**), eupatorin (**68**), and genkwanine (**69**) in both ultrasonic extracts. The authors isolated phenolic acids from *S. deserta* and found that the flowers contain a higher amount of caffeic (**1**), rosmarinic (**2**), and ferulic (**23**) acids [42]. High-performance thin-layer chromatography (HPTLC) was successfully applied to the qualitative and quantitative analyses of caffeic (**1**), rosmarinic (**2**), oleanolic (**70**), and ursolic (**71**) acids in different parts of *S. deserta* [50]. The results showed that the content of each of these components was higher in flowers than in roots, stems, and leaves. Other authors for the first time [51] extracted salvisertin A (**72**), dichroanone (**73**), and sugiol (**74**) (Figure 6).

Based on the conducted study on eight species of sage, it is noted that the compositional content depends on the geographical location, plant part, extraction method, conditions, and chosen solvent. It has also been established that the literature provides limited distribution data; for example, data on the compositional analysis of *S. virgata* are only available for Turkey and Iran, for *S. macrosiphon* only for Iran, for *S. dumetorum* only in the European part, and for *S. deserta* only for Kazakhstan and China, whereas some of these species grow in Central Asia, the European part of Russia, and European countries. Furthermore, there is a lack of data in the literature regarding the compositional content of extracts from *S. trautvetteri*. Thus, this review indicates the prospects of the in-depth study of these species for the needs of the pharmaceutical industry.

## 5. Volatile Organic Compounds (VOCs)

Essential oils (EOs) contain VOCs of various functional groups. The composition of the EOs is extensive, making the study of their compositional content highly relevant.

This review analyzes the available literature dedicated to studying the compositional content of the EOs from eight species of the genus *Salvia*. The analysis revealed that quantitative data on abundant constituents, different morphological forms, and distribution ranges are most frequently available, while for some plants, compositional analysis data are completely absent. Additionally, studies on individual plant species have identified differences in their chemical composition depending on their distribution range. Therefore, the variability in the chemical composition of plants may be influenced by ecological factors and their habitat.

Thus, gathering comparative data on the chemical composition of certain species of *Salvia* is essential for selecting plants dominant in components necessary for biological activity in future research.

### 5.1. VOCs from S. virgata Essential Oil

**Figure 7:**
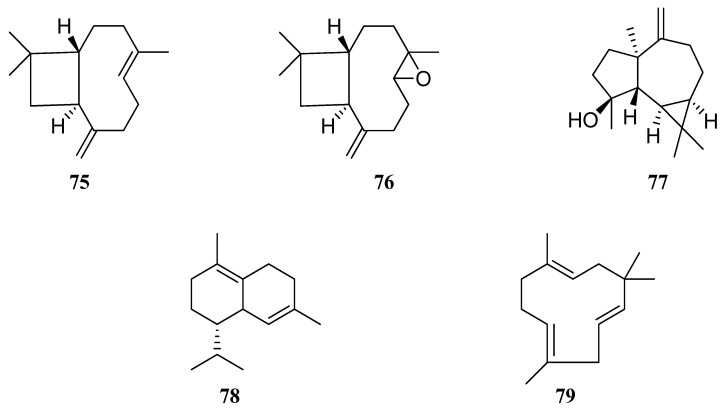
Chemical structures of sesquiterpenoids found in *S. virgata* essential oil.

Comparative data on the compositional content of VOCs in *S. virgata* EO are presented in Table 4. The major constituents of the *S. virgata* EO presented sesquiterpenoids such as β-caryophyllene (**75**) (3.1–48.12%), caryophyllene oxide (**76**) (6.03–34.4%), spathulenol (**77**) (0.17–25.6%), δ-cadinene (**78**) (0.15–23.3%), and α-humulene (**79**) (0.50–10.88%) (Figure 7).

**Figure 8:**
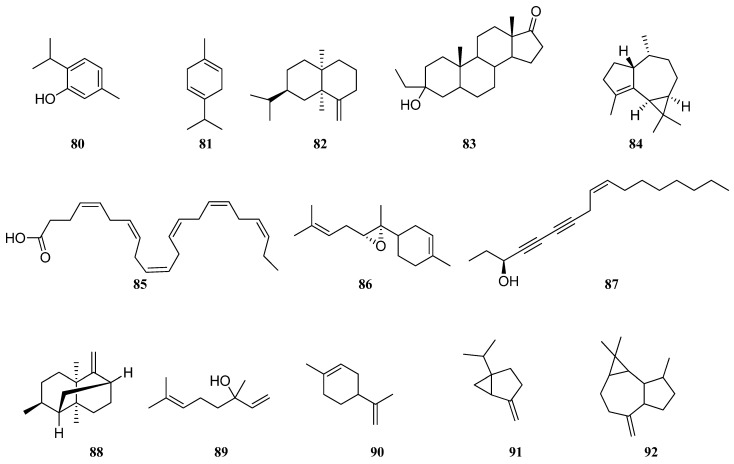
VOCs found in *S. virgata* essential oil.

In addition, only *S. virgata* EO from Krym [58] was obtained from leaves, all other studies were conducted on the aerial part of *S. virgata;* the results are inconsistent. Comparing *S. virgata* samples from Iran, it can be seen that chemical composition of essential oil from one country is quite different. Golparvar et al. [52] and Morteza-Semnani et al. [53] reported that caryophyllene oxide (**76**) at 30.23 and 34.4% was the most abundant component, followed by spathulenol (**77**) at 0.17 and 25.6%, α-humulene (**79**) at 0.95 and 0.1%, thymol (**80**) at 0.75 and 0.2%, β-caryophyllene (**75**) at 22.6 and 3.1%, and γ-terpinene (**81**) at 1.12 and 0.3%, respectively. On the other hand, in a study conducted by Esmaeli et al. [16], caryophyllene oxide (**76**) was not detected. However, valeranone (**82**), followed by 3-Ethyl-3-hydroxyandrostan-17-one (**83**), α-gurjunene (**84**), doconexent (**85**), *cis*-Z-α-bisabolene epoxide (**86**), falcarinol (**87**), and seychellene (**88**) were detected only in this study [16]. *S. virgata* EO from Krym, rich in terpenoids, presented linalool (**89**) and limonene (**90**) [58]. Sabinene (**91**) at 21.2% and aromadendrene (**92**) at 15.2% were the main constituents of *S. virgata* EO [57] (Figure 8).

### 5.2. VOCs from S. macrosiphon Essential Oil

Comparative data on the component composition of VOCs of *S. macrosiphon* EO are presented in Table 5.

**Figure 9:**
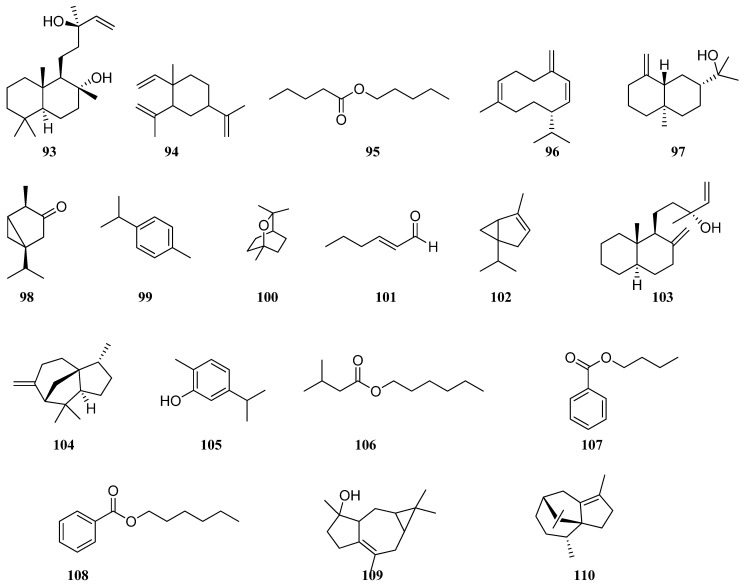
VOCs found in *S. macrosiphon* essential oil.

Salimpour et al. [61] reported that in *S. macrosiphon* EO, 67 constituents were found, representing 97.28% of the total components; the main components were sclareol (**93**) (8.60%), spathulenol (**77**) (5.86%), β-elemene (**94**) (5.44%), hexyl n-valerate (**95**) (4.84%), germacrene D (**96**) (4.31%), and β-eudesmol (**97**) (3.88%). In other studies, the amount of these compounds was lower or none at all [16,60,62,63]. Linalool (**89**) (19.0–54.8%) was the major constituent of *S. macrosiphon* EO [16,22,62]. Sixty VOCs were identified in *S. macrosiphon* leaves [60]. Among them, representing 90.9% of the total components, α-thujone (**98**) (17.84%), γ-terpinene (**81**) (14.75%), β-caryophyllene (**75**) (9.92%), p-cymene (**99**) (7.25%), 1.8-cineole (**100)** (5.82%), 2-hexenal (**101)** (5.81%), α-thujene (**102**) (4.28%), limonene (**90**) (2.73%), and germacrene D (**96**) (2.14%) dominated. Manool (**103**) (27.3%) was introduced as a principal component of *S. macrosiphon* EO by Karminik et al. [62]. The major constituents of the *S. macrosiphon* EO obtained from Fars province (Iran) were presented as sesquiterpenes and identified as linalool (**89**) (19.0%), β-elemene (**94**) (13.33%), β-cedrene (**104**) (13.33%), carvacrol (**105**) (9.96%), thymol (**80**) (8.73%), and hexyl isovaleriate (**106**) (5.06%) [22]. The twenty-nine components in the *S. macrosiphon* leaf EO from Kerman province (Iran) were dominated by butyl benzoate (**107**) (49.16%), *n*-hexyl benzoate **108** (7%), isospathulenol (**109**) (4.8%), and cyperene (**110**) (4.1%) (Figure 9) [63]. Results reported in literature clearly highlighted differences among VOC patterns of the *S. macrosiphon* EO growing in Iran.

### 5.3. VOCs from S. sclarea Essential Oil

Comparative data on the component composition of VOCs from *S. sclarea* EO are presented in Table 6.

**Figure 10:**
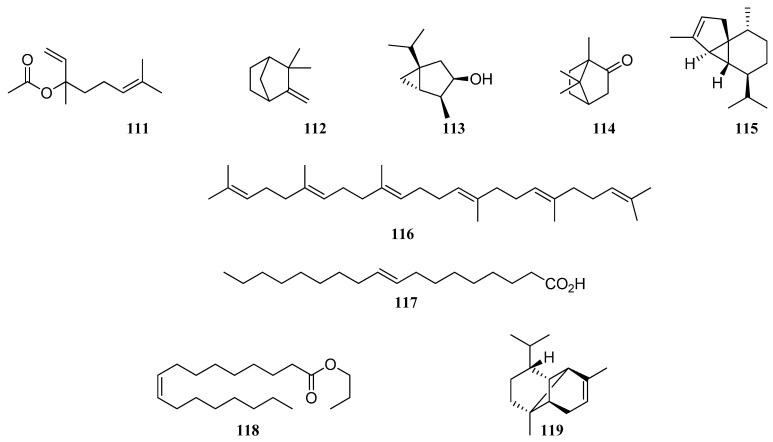
VOCs found in *S. sclarea* essential oil.

According to available literature, linalyl acetate (**111**) and linalool (**89**) were the major constituents of *S. sclarea* EO, with ranges from 4.7 and none [59], 20.5 and 13.78–26.20 [16,70], 34.62 and 17,67 [69], 39.2 and 12.5 [67], 49.1 and 20.6 [66] to 59.3 and 11.3 [68], and 18.8 and 14.9% [71], respectively. *S. sclarea* EO compositions from Uzbekistan, Iran, India, Bulgaria, Tadjikistan, Slovakia, Georgia, and Italy obviously related to a linalool/linalyl acetate-rich chemotype. However, in *S. sclarea* from Lithuania [64] and Poland [65], linalool (**89**) and linalyl acetate (**111**) were not detected, so these compounds obviously related to different chemotypes. Moreover, the major compounds of *S. sclarea* EO from Poland were camphene (**112**) (22.36%), followed by thujol (**113**) (12.31%) and camphor (**114**) (2.74%) [65], while *S. sclarea* EO from Lithuania was rich in carophyllene oxide (**76**) (14%), followed by β-cubebene (**115**) (12.3%), squalene (**116**) (10.9%), and germacrene D (**96**) (9.6%) (Figure 10) [64]. In *S. sclarea* EO from Uzbekistan, the major compounds were 9-octadecenoic acid (**117**) (6.9%), followed by *n*-butyloctadecenoate (**118**) (5.7%) and linalyl acetate (**111**) (4.7%) [59]. In addition, six sesquiterpenoids such as β-caryophyllene (**75**), carophyllene oxide (**76**), δ-cadinene (**78**), germacrene D (**96**), β-eudesmol (**97**), and α-copaene (**119**) were found in the *S. sclarea* EO with varying percentages. Esmaeli et al. [16] have found the highest similarity in *S. sclarea* and *S. macrosiphon* species from Iran, with regards to high amounts of linalool (**89**), germacrene D (**96**), and β-caryophyllene (**75**).

### 5.4. VOCs from S. dumetorum Essential Oil

Comparative data on the component composition of VOCs of *S. dumetorum* EO are presented in Table 7. Data on the chemical composition of *S. dumetorum* EO in the available literature are quite limited. We were able to find studies of the component composition of *S. dumetorum* EO using gas chromatography–mass spectrometry (GC–MS) only in four countries.

**Figure 11:**
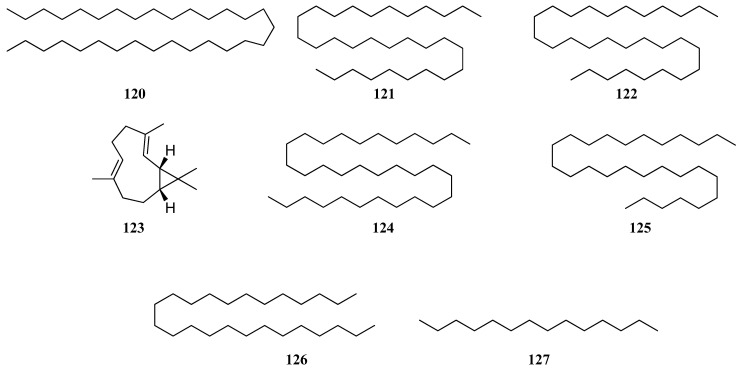
VOCs found in *S. dumetorum* essential oil.

During the analysis of these studies, significant differences in compositions were found, e.g., major constituents in *S. dumetorum* from Lithuania [64] were caryophyllene oxide (**76**) and squalene (**116**) (20.1%), from Poland [65] were camphene (**112**) (17.26%) and thujol (**113**) (8.42%), from Kazakhstan [72] were β-caryophyllene (**75**) (13.21%), caryophyllene oxide (**76**) (11.16%), and spathulenol (**77**) (10.75%), and from Ukraine [33] were hentriacosane (**120**) (15.3%), tritiacontane (**121**) (12.9%), and nonacosane (**122**) (5.3%). Sesquiterpenes, such as α-humulene (**79**) and α-gurjunene (**84**), germacrene D (**96**), and bicyclogermacrene (**123**), were first identified in *S. dumetorum* EO from Kazakhstan [72]. Lithuanian *S. dumetorum* [64] EO was characterized by different classes of VOCs, such as diterpenes, sesquiterpenes, triterpenes, hydrocarbons, and monoterpenes. EO from Poland [65] belongs to the camphene/camphore chemotype of *S. dumetorum* and contains monoterpenes. Koshovyi et al. reported that *S. dumetorum* EO from Ukraine [33] was dominated by hydrocarbons (hentriacosane (**120**), tritiacontane (**121**), nonacosane (**122**), dotriacontane (**124**), heptacosane (**125**), pentacosane (**126**), and tetradecane (**127**)) (Figure 11).

### 5.5. VOCs from S. verticillata Essential Oil

Comparative data on the component composition of VOCs of *S. verticillata* EO are presented in Table 8. The qualitative and quantitative composition of *S. verticillata* EO chemical constituents was completely different in all studies.

**Figure 12:**
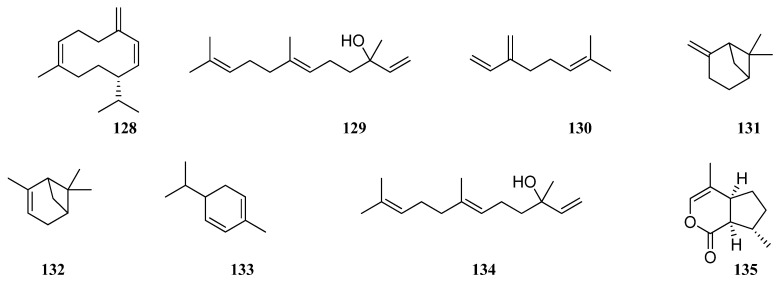
VOCs found in *S. verticillata* essential oil.

For instance, germacrene B (**128**) and nerol (**129**) are the most abundant compounds in *S. verticillata* from Lithuania [64] and were not detected in any of the other *S. verticillata* studies [57,73,75,77], whereas myrcene (**130**), bicyclogermacrene (**123**), and β-pinene (**131**) were also present there with different quantitative composition. Thirty-nine components representing 83.8% of the *S. verticillata* EO were identified by GC–MS; the major constituents were spathulenol (**77**) (31.0%), α-pinene (**132**) (8.2%), and limonene (**90**) (4.1%) [73]. Dehaghi et al. [77] identified 59 components in the *S. verticillata* EO representing 97.67% of the total oil, which was characterized by a high amount of sesquiterpenes and monoterpenes, among them β-caryophyllene (**75**) (24.40%), β-phellandrene (**133**) (9.08%), α-humulene (**79**) (8.61%), bicyclogermacrene (**123**) (6.32%), spathulenol (**77**) (5.89%), and β-pinene (**131**) (5.00%). Inverse results were obtained in *S. verticillata* EO from Serbia [76], where an increase in the content of β-phellandrene (**133**), 43.9–55.5%, and α-pinene (**132**), 1.9–21.1%, leads to a decrease in the content of β-caryophyllene (**75**), 0.3–0.9%, and β-pinene (**131**), 3.0–3.6%, depending of the region, respectively (Figure 12). According to the results, Ukranian *S. verticillata* [75] does not contain β-caryophyllene (**75**) and β-pinene (**131**) at all, while other *S. verticillata* samples show varying levels of them. Giuliani et al. investigated the chemical profile of *S. verticillata* EO obtained from aerial parts [73]. The results showed that the EO was dominated by sesquiterpenes, followed by monoterpenes. As reported, principal compounds were germacrene D (**96**), bicyclogermacrene (**123**), β-caryophyllene (**75**), α-humulene (**79**), and β-phellandrene (**133**). Tomou et al. [57] studied the chemical composition of cultivated and wild-growing EO. The results presented qualitative and quantitative differences between the two samples. Cultivated *S. verticillata*’s main chemical constituents were nerolidol (**134**) (35.0%), germacrene D (**96**) (11.5%), and β-pinene (**131**) (6.1%), whereas 4aα,7α,7aα-nepetalactone (**135**) (51.4%), 1.8-cineole (**100**) (18.4%), and caryophyllene oxide (**76**) (6.0%) were the dominating components in wild-growing *S. verticillata*.

### 5.6. VOCs from S. aethiopis Essential Oil

Comparative data on the component composition of VOCs of *S. aethiopis* essential oil are presented in Table 9.

**Figure 13:**
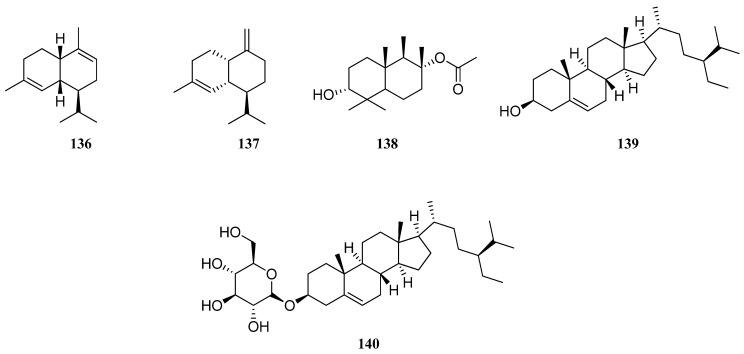
VOCs found in *S. virgata* essential oil.

Damyanova et al. investigated the chemical composition of flowers and leaves of *S. aethiopis* from Bulgaria [79]. It can be seen that in both EOs twenty components were identified. Results showed that there is no big difference in the quantitative content of constituents. Thus, *S. aethiopis* flower EO was rich in germacrene D (**96**) (29.37 %) and β-caryophyllene (**75**) (23.55%), while leaf EO contained more α-copaene (**119**) (17.24%), β-cubebene (**115**) (9.71%), α-humulene (**79**) (6.79%), and δ-cadinene (**78**) (6.69%) than flower. The authors noted that Bulgarian *S. aethiopis* EO belongs to a germacrene D and β-caryophyllene chemotype. This statement is strongly confirmed by other studies. The principal constituents of *S. aethiopis* EO are sesquiterpenes, mainly germacrene D (**96**) and β-caryophyllene (**75**) [57,78,79]. High α-copaene (**119**) (33.4%), β-elemene (**94**) (7.3%), and several new compounds, presented by α-muurolene (**136**) (7.2%) and γ-muurolene (**137**) (10.3%) amounts were noted in *S. aethiopis* EO from Serbia [76], in contrast to previous studies. White et al. [80] isolated one new (**138**) and three known compounds from Kazakhstani *S. aethiopis* EO, identified as spathulenol (**77**), β-sitosterol (**139**), and β-sitosterol-3-O-β-d-glucoside (**140**) (Figure 13).

### 5.7. VOCs from S. deserta Essential Oil

Comparative data on the component composition of VOCs of *S. deserta* are presented in Table 10.

**Figure 14:**
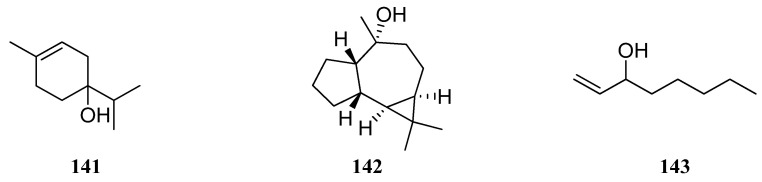
VOCs found in *S. deserta* essential oil.

EO obtained from *S. deserta* by the hydrodistillation of leaves and flowers was analyzed with a GC–MS system [42]. EO derived from *S. deserta* flowers contained at most 72 compounds; β-phellandrene (**133**) (29.74%) dominated, followed by 4-terpineol (**141**) (10.91%) and ledol (**142**) (6.98%). The principal components of *S. deserta* leaf EO were ledol (**142**) (8.36%), caryophyllene oxide (**76**) (5.99%), and 1-octen-3-ol (**143**) (4.98%) (Figure 14). However, *S. deserta* EO from Poland [65] had a different chemical composition compared to *S. deserta* from China. The principal constituents of *S. deserta* were α-pinene (**132**) (35.5%), followed by β-pinene (**131**) (13.02%), limonene (**90**) (7.45%), and camphene (**112**) (4.01%) [65].

According to the chemotaxonomic study of the genus *Salvia* conducted by Koshovyi et al. [33], four main clusters of species were determined. This analysis showed that *S. aethiopis* and *S. sclarea*, presenting apigenin and hispidulin, belong to one taxonomic group, whereas *S. dumetorum* is distinguished by quercetin and rutin. Esmaeili et al. [16] found high similarity between two species of the *Salvia* genus, *S. sclarea* and *S. macrosiphon*, in terms of linalool, germacrene D, and β-caryophyllene.

The differences between the *Salvia* L. species are based on their geographical distribution, ecology, and chemical composition. These differences may reflect adaptation to local conditions and evolutionary features. At the same time, the lack of information on the *S. trautvetteri* may be due to lesser knowledge on it and its limited use, which reduces the level of scientific research on this plant.

## 6. Biological Activity

**Figure 15:**
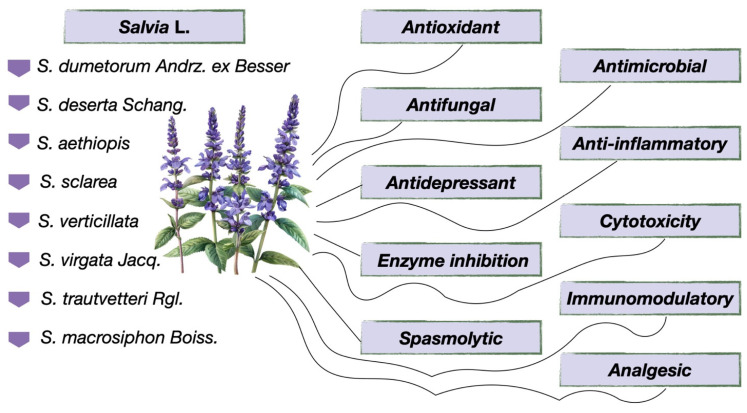
Biological activity of *Salvia* spp. plants.

Plants are a source of a wide variety of BASs that are extensively used in medicine, cosmetics, and the food industry. The biological activity of plants in the *Salvia* genus is attributed to their ability to synthesize diverse metabolites, such as phenolic compounds, terpenoids, alkaloids, and others, which in turn exhibit various pharmacological effects, including antioxidant, anti-inflammatory, antimicrobial, anticancer, and others (Figure 15). Research on the biological activity of plants involves analyzing the effects of secondary metabolites on cell models, animals, and humans, as well as the mechanisms of their action at the biological level. These studies are aimed at exploring the potential use of plant extracts in medicine.

Due to the rather extensive chemical composition of plants in the *Salvia* genus, we conducted an analysis of the existing literature on the biological activity of eight species that grow in Kazakhstan (Table 11). The gathered data will help specialists in this field identify new, yet unexplored directions for studying the biological activity of *Salvia* spp. and determine the potential use of these plants as pharmaceutical substances and medicinal products.

The data summarized in Table 11 presents a detailed exploration of the biological activities of the various eight *Salvia* species.

Antioxidant activity showed that *Salvia* spp. plants exhibited varying antioxidant potential across different extracts. For example, *S. virgata.* methanol extract had an IC_50_ for DPPH scavenging of 0.2 mg/mL [17], making it one of the most potent scavengers among tested extracts. Furthermore, *S. virgata* EO exhibited moderate antioxidant activity with IC_50_ values of 1.98 mg/mL (DPPH), 0.75 mg/mL (ABTS), and 0.39 mg/mL (CUPRAC) [59], suggesting that EO might be a promising source of antioxidant compounds. In comparison, the ethanolic extract of *S. virgata* showed a much higher IC_50_ for DPPH 291.58 μg/mL, indicating a less potent antioxidant effect than the methanolic extract [20]. *S. macrosiphon* showed potent antioxidant activity, with the highest radical scavenging activity in its root extract, IC_50_ = 10.9 μg/mL [16], and significant activity in the FRAP assay, 17.4 ± 2.3 μM eq-QE/g dry weight [21]. *S. sclarea* and *S. verticillata* also demonstrated significant antioxidant properties in various assays such as DPPH, ABTS, and FRAP. The antioxidant activity of these species varied depending on the extract type, with the methanolic extract consistently showing higher scavenging potential than other solvents. For example, the methanolic extract of *S. sclarea* had an IC_50_ of 190.74 ± 5.7 μg/mL in the DPPH assay [26], suggesting moderate antioxidant capacity. *S. verticillata* was found to have strong antioxidant activity, especially in the DPPH assay, IC_50_ = 27.36 ± 0.32 μg/mL, and ABTS, IC_50_ = 13.40 ± 0.10 μg/mL [27]. *S. aethiopis* exhibited varying antioxidant capabilities, with the methanolic extract showing moderate DPPH scavenging, IC_50_ = 123.37 ± 8.05 μg/mL, compared to other species, but still significant in some assays [26]. The same data were found for the *S. sclarea* EO in the DDPH assay, IC_50_ = 123 ± 0.99 μg/mL [68]. Overall, *S. virgata* and *S. macrosiphon* appear to be the strongest antioxidants, particularly in the methanolic extract form. However, the EO of *S. virgata* stands out for its exceptional performance in cupric ion reducing antioxidant capacity (CUPRAC) and ferric reducing antioxidant power (FRAP) assays.

Antimicrobial activity. *S. virgata* EO demonstrated broad-spectrum antimicrobial activity, particularly against *Staphylococcus epidermidis*, *Penicillium funiculosum*, and *Escherichia coli*, indicating strong antimicrobial properties. Its ethanolic extract was moderately active against *Staphylococcus aureus* and *E. coli*, with an MIC of 0.312 mg/mL for both [20]. The EO showed moderate antimicrobial activity against *S. aureus* and *Candida albicans* [81]. *S. macrosiphon* exhibited strong antimicrobial potential, with MIC values as low as 1.25 mg/mL for *S. aureus*, *S. epidermidis*, and *Bacillus subtilis*, making it highly effective in combating Gram-positive bacteria [83]. The *n*-hexane fraction also showed activity against *S. aureus* and *E. coli*, suggesting that non-polar extracts might be particularly useful for targeting bacterial pathogens. *S. sclarea* showed varied antimicrobial effectiveness, with MIC values ranging from 1.25 to 10 mg/mL for different bacterial strains. Notably, the methanolic extract was highly active against *S. aureus* and *S. epidermidis*, with MIC values of 1.25 mg/mL [26]. *S. verticillata* demonstrated effective antimicrobial activity against a range of bacteria, including *S. aureus* MIC = 1.25 mg/mL and *E. coli* MIC = 2.5 mg/mL [27]. The species’ essential oil showed good antimicrobial properties, particularly against biofilm-forming *Pseudomonas fluorescens*. *S. aethiopis* displayed moderate to high antimicrobial activity against Gram-positive and Gram-negative bacteria, with MICs ranging from 1.25 to 5 mg/mL [27] depending on the strain. The EO of *S. aethiopis* was especially effective against *S. aureus* bacteria. *S. dumetorum* ethanolic extracts in different concentrations have promising antimicrobial activity towards *S. aureus*, *B. subtilis*, and *E. coli* [91]. In comparison, *S. macrosiphon* and *S. virgata* exhibited some of the most potent antimicrobial activities, especially for the methanolic extract and EO, respectively. *S. sclarea* and *S. verticillata* also showed promising antimicrobial potential, particularly against Gram-positive bacteria.

Cytotoxicity. The ethanolic extract of *S. virgata* demonstrated significant cytotoxicity against the MDA-MB-231 breast cancer cell line with an IC_50_ of 0.118 mg/mL, while showing minimal cytotoxic effects on the L929 cell line, indicating a selective cytotoxic profile. The EO also demonstrated moderate cytotoxicity [20]. *S. macrosiphon* EO exhibited potent cytotoxic activity on MCF-7, MDA-MB-231, and T47D cell lines, with an IC_50_ of <0.15 μg/mL, whereas its total extract showed moderate activity (IC_50_ < 100 μg/mL) [22]. The hydromethanolic extract also demonstrated cytotoxicity, especially in the A549, MCF-7, and MDA-MB-231 cell lines, with IC_50_ values ranging from 10.24 to 20.89 μg/mL [85]. *S. sclarea* showed weak cytotoxic effects, as evidenced by its lack of significant cytotoxicity on MCF-7 and MDA-MB-231 cell lines, although slight increases in viable cells were observed [32]. *S. verticillata* exhibited variable cytotoxicity across different cancer cell lines, with the methanolic extract showing IC_50_ values ranging from 72.8 ± 1.9 (SH-SY5Y) to 166.3 ± 2.4 μg/mL (MCF-7), indicating moderate cytotoxic potential [95]. *S. dumetorum* hexane extract at a concentration of 10 μg/mL exhibited a cell proliferation rate of 27.8 ± 1.0% on the MCF-7 breast cancer cell line, whereas the chloroform extract demonstrated a cell proliferation activity of 41.1 ± 0.9% on the A431 skin cancer cell line [90]. Overall, *S. macrosiphon* and *S. virgata* exhibited the strongest cytotoxic effects, particularly their EOs, which can be attributed to their bioactive compounds. *S. aethiopis* also demonstrated some cytotoxicity, albeit at higher concentrations.

Enzyme Inhibition. *S. virgata* demonstrated enzyme inhibition across several pathways. Its methanolic extract exhibited an inhibition of α-glucosidase (70.2%), α-amylase (81.2%), acetylcholinesterase (AChE, 16.45%), and butyrylcholinesterase (BChE, 25.98%) in a concentration-dependent manner [18]. The EO showed high BChE inhibition IC_50_ = 0.60 mg/mL, but weaker activity against AChE and α-amylase [59]. *S. sclarea* exhibited moderate to strong enzyme inhibition in various assays. Its methanolic extract showed AChE inhibition at 47% IC_50_ = 200 μg/mL, while its ultrasonic extract demonstrated moderate urease inhibition [28]. *S. macrosiphon* demonstrated AChE inhibition with IC_50_ values around 0.169 μg/mL, with no activity observed for BChE inhibition at concentrations up to 500 μg/mL [22]. This suggests that *S. macrosiphon* may be more effective in targeting cholinergic pathways. *S. verticillata* exhibited moderate enzyme inhibition, particularly against AChE and BChE, but at relatively high concentrations compared to other species. In terms of enzyme inhibition, *S. virgata* emerged as a leader due to its broad spectrum of activity across several enzymes, followed by *S. sclarea*, which also showed significant potential, especially in cholinergic pathways.

The relationship between the phytochemical composition of plants and the biological activity of their extracts is a very important topic of study, as it is these substances that determine the potential of plants in medicine and pharmaceuticals. Each of the phytochemical compounds has unique properties that can exert a wide range of biological effects on the human body. These compounds can act both synergistically and in isolation, enhancing or weakening the overall effect of the extract. Thus, a complex extract of a plant may show more pronounced activity than the individual components due to their interaction with each other. For example, in some plants, flavonoids and terpenes can enhance anti-inflammatory effects and alkaloids can stimulate tissue regeneration.

Recent studies have highlighted high antioxidant, anti-inflammatory and antimicrobial activity of phenolic compounds, mainly flavonoids and phenolic acids [101,102,103,104,105,106]. Phenolic compounds play a key role in antioxidant mechanisms; they remove free radicals and prevent their interaction with the DNA, increase the activity of antioxidant enzymes and detoxification enzymes, and thus reduce oxidative damage, eliminate potential mediators of inflammation and carcinogens, and stimulate cytoprotective mechanisms. For example, e.g., the flavonoid quercetin (**16**) found in *S. sclarea* and *S. dumetorum* or other derivates with 3′,4′-dihydroxy substituents in ring B and conjugation between rings A and B, such as protocatechuic acid (3,4-dihydroxy benzoic acid) (**52**) and protocatechuic aldehyde (**61**) (3,4-dihydroxybenzaldehyde), show pronounced antioxidant activity [101,102]. The presence of apigenin (**21**) was demonstrated in *S. aethiopis*, *S. sclarea*, and *S. verticillata* and showed good antioxidant [103,104], antimicrobial [105], anti-inflammatory, antifungal, and antiparasitic activity [106].

From the class of phenolic acids, one of the most powerful secondary metabolites of plants is rosmarinic acid (**2**), which is found in almost all species of the genus *Salvia* in large quantities. The presence of a phenolic group in the molecule determines its biological activity. Rosmarinic acid (**2**) neutralizes free radicals and reduces the level of lipid peroxides, which helps prevent cellular damage and maintain the integrity of cell membranes, inhibits the activity of cyclooxygenase (COX), an enzyme involved in the synthesis of prostaglandins—mediators of inflammation, inhibits the growth of various pathogenic microorganisms, including bacteria the *Staphylococcus* spp. and *E. coli* and fungi *C. albicans*, inhibits the proliferation of tumor cells and stimulates apoptosis (programmed cell death), which makes it a potential candidate for use in the treatment of various forms of cancer, and has a positive effect on blood sugar levels, reducing its concentration and improving tissue sensitivity to insulin [107]. Chlorogenic (**42**) and p-coumaric (**43**) acid found in *S. aethiopis* and *S. verticillata* showed pronounced antimicrobial, antioxidant, and anti-inflammatory action. Investigations into the antimicrobial activity mechanism of *p*-coumaric acid (**42**) unveiled a dual mechanism: it disrupts bacterial cell membranes and can bind to the phosphate anion in the DNA double helix, thereby intercalating into the DNA groove and affecting replication, transcription, and expression [108]. The phenolic hydroxyl structure readily reacts with free radicals and forms hydrogen free radicals with anti-oxidant effect, eliminating hydroxyl radicals and superoxide anions as well as exhibiting a strong anti-oxidant effect [109]. Terpenes α-pinene (**132**) and β-pinene (**131**), detected in seven species discussed in this review with the exception of *S. dumetorum* which contain only α-pinene (**132**), were demonstrated to have antimicrobial and antifungal effects [110].

Thus, secondary metabolites of plants influence their therapeutic properties, including antioxidant, anti-inflammatory, antibacterial, and antitumor activities. Studies also show that the combination of several phytochemicals can enhance biological activity, which opens new perspectives for the development of effective phytotherapeutic drugs.

Across the various biological activities tested, *S. virgata* and *S. macrosiphon* consistently demonstrated the strongest and most diverse bioactivities, particularly in terms of antioxidant, antimicrobial, and cytotoxic properties. *S. sclarea* and *S. verticillata* also exhibited significant bioactivity, especially in enzyme inhibition and antimicrobial testing, but with somewhat lower efficacy in certain assays compared to *S. virgata* and *S. macrosiphon*. *S. aethiopis*, while showing promise in specific assays (e.g., antioxidant), had relatively weaker overall bioactivity. Thus, species like *S. virgata* and *S. macrosiphon* may hold greater potential for pharmaceutical and therapeutic applications due to their broader range of bioactivities and potency.

## 7. Materials and Methods

### 7.1. Strategy for Literature Search

The information presented in this paper was gathered through an extensive literature search using various computerized databases such as ^®^ScienceDirect, ^®^PubMed, ^®^SciFinder, ^®^Web of Science, ^®^Scopus, and ^®^GoogleScholar. Additional data were sourced from academic dissertations, theses, and relevant books in plant sciences, State Pharmacopoeias of different countries, and ethnobotany. The search followed the guidelines set by the Preferred Reporting Items for Systematic Reviews and Meta-Analyses (PRISMA) statement [111]. Relevant keywords such as “botany”, “Salvia dumetorum”, “Salvia macrosiphon”, “Salvia trautvetteri”, “Salvia aethiopis”, “Salvia deserta”, “Salvia virgata”, “Salvia verticillata”, “enzyme inhibition”, “cytotoxicity”, “chemical compounds”, “antimicrobial activity”, “anti-inflammatory activity”, “survey”, “phytochemistry”, “antioxidant”, “antiparasitic”, “essential oil”, “extraction methods”, “antidiabetic”, and “ethnopharmacological aspects” were used interchangeably in the search process.

### 7.2. Data Mining to Generate the Inventory/Data

The inclusion criteria were as follows: (1) literature sources should use recognized scientific methods and techniques for studying the botanical composition, phytochemistry, and biological activity of plants, as well as those where the results were obtained in controlled and reproducible experiments; (2) review only those species of the genus *Salvia* that are widely distributed in Kazakhstan and for which detailed data on morphology, classification, ecology, and distribution in Kazakhstan are available; (3) the study should not be focused on species of the genus *Salvia* that are not widespread in Kazakhstan or do not have a significant presence in the region; and (3) the study should be based on the following criteria.

On the other hand, the exclusion criteria were as follows: (1) *Salvia* species that are not widespread in Kazakhstan or have no significant presence in the region; (2) studies that do not describe clear methodological approaches or do not provide sufficient data to reproduce the experiments and validate the results; (2) articles without scientific names of plants; and (3) studies that do not correspond to the main topics of the article—botany, phytochemistry, or biological activity of *Salvia* plants.

Data were collected with the help of the library staff of Karaganda Medical University. In the search system, plant species with only the generic name *Salvia* were omitted in this paper. The task was completed by the first author and confirmed by the second author. Plant parts, methods of their extraction, the phytochemical composition of the volatile part and extracts, and data on biological activity were listed from each relevant article.

## 8. Conclusions

Extensive research on the chemical composition and pharmacological activity of plants from the *Salvia* genus has been conducted at scientific centers in several countries (Italy, China, Russia, Iran, Lithuania, Turkey, Georgia, Tajikistan, Kazakhstan, etc.). These studies have shown that the chemical composition of these plants varies significantly due to abiotic and biotic factors, as well as extraction methods. Variability in the chemical composition (both qualitative and quantitative) of plant extracts or EO can lead to substantial differences in their pharmacological activities. The variation in biological activity and chemical composition may be due to differences in the extraction method, solvent polarity, or plant material used. The further optimization of the extraction procedure and testing with other solvents or bioactive compounds may introduce new avenues of research and open new possibilities for further investigation. Currently, not all species of *Salvia* have been studied for their chemical composition and biological activity. Many of the identified metabolites have important biological roles, both in plants as well as for human organisms.

The high content of flavonoids and phenolic compounds in the *Salvia* species indicates the prospects for an in-depth study of these species for the needs of the pharmaceutical industry.

In conclusion, the antioxidant activities of selected *Salvia* species make them a valuable subject for the pharmaceutical and food industries. Some species, such as *S. dumetorum*, *S. deserta*, and *S. aethiopis* have been poorly investigated earlier, and they are therefore of interest, primarily in the search for promising new sources of natural antioxidants.

## Figures and Tables

**Table 1:** Morphological characteristics of *Salvia* species distributed in Kazakhstan [3].

Morphological Characteristics	*S. aethiopis*	*S. sclarea*	*S. dumetorum*	*S. deserta*	*S. trautvetteri*	*S. macrosiphon*	*S. virgata*	*S. verticillata*
Stem	Short, pyramidal branched, ribbed, densely pubescent.	Longer than inflorescence, pubescent with curly hairs.	Short and rarely pubescent at the base with a thick rosette of basal leaves.	In the upper part, longer than the inflorescence, densely pubescent.	Covered with glandular and simple spaced hairs.	Densely hairy, emerges from woody root.	Shorter than inflorescences, leafy, pubescent below with multicellular hairs.	Numerous, densely pubescent multicellular hairs.
Leaves	Basal ovate, oblong heart-shaped, sharp or blunt, town-dentate at the edges, sometimes lobed. Stem sessile, oblong-ovate, sharp- or blunt-toothed.	Basal ones are smaller, curl early and dry out. Stem ovate or ovate- oblong, sharp or blunt, wrinkled, long-leaved.	Basal numerous, oblong or barely ovate, slightly heart-shaped at the base, blunt. Stem often larger than basal, short tiled or sessile.	Basal leaves are small, drying early. Stem significantly smaller, ovate or at the apex long thinly drawn, sessile or very short-leaved.	Basal pinnately dissected, oblong or oblong-elliptical in outline, 5–7 cm long, 3–4 cm wide. Stem reduced, short-leaved, upper sessile.	Basal oblong-ovate, twofold and sharply gnawed-serrated along the edge, softly pubescent along the veins on both sides.	Basal elliptical, oblong or ovate-oblong, blunt or rounded along the edge of the town, twofold town. Stem lanceolate, sessile, almost lobed along the edge, pubescent.	Basal on petioles of equal plates, stem heart-ovate, 4–13 cm long, 3–10 cm wide, sharp, the edges of the leaf blade are town-like.
Inflorescence	Pyramidal panicle, branches with 4–6 close, 6–10-flowered false whorls.	Paniculate branched, with 2–6 floral false whorls.	Simple or with one or two pairs of lower branches, with 5–10 false 4–6-flowered whorls.	With one or two pairs of simple branches, with 20–25 false 4–6-flowered whorls.	A simple or branched brush, glandularly pubescent pedicels, 1–1.5 cm long, 2–3 in whorls.	Spreading panicle, with 3–6 color whorls spaced.	Long, with 2–3 pairs of protruding, long rod-shaped branches, with 6-flowered false whorls.	Simple or with one or two pairs of long branches, of 20–40-flowered false whorls.
Calyx	10–16 mm long, tubular-bell-shaped, two-lipped, with five well-developed acutely pointed teeth.	10–12 mm long.	7 mm long, upper lip shorter than lower, semicircular, lower— two-toothed.	7 mm long, upper lip shorter than lower, rounded, lower—two-toothed.	Two-lipped, bell-shaped, green, sometimes painted in purple tones, three-dentate.	15–20 mm long, narrow-tube with protruding veins.	8–9 mm long, upper lip shorter than lower, with three short close teeth, lower lip three-dentate.	Tubular, often lilac.
Corolla	White, 12–22 mm long.	Pinkish, white or lilac.	Dark blue, 15–18 mm long.	Dark purple, 10–16 mm long.	White, occasionally bluish, 30–40 mm long.	White, 20–25 mm long.	Pale purple, 18–25 mm long.	Purple, sometimes white, 10–13 mm long.
Fruits	Ellipsoidal trihedral, 2–2.5 mm long, greenish-brown.	Ellipsoidal, 2–3 mm long, reticular-wrinkled, brown.	Trihedral spherical, 2 mm in diameter, dark brown.	Trihedral spherical, 1.5–2 mm in diameter, dark brown.	Trihedral spherical, brown, with stripes.	Naked, round-ovoid, brownish.	Spherical, 2 mm diameter, smooth trihedral, dark brown.	Round elliptical, smooth, 1.5–5 mm long, from light brown to dark.
Habitat	In the steppes and on the meadow slopes of the steppe mountains.	On gravelly and rocky mountain slopes, in gorges and valleys.	In the steppes and on dry steppe meadows.	In the steppe zone, along the mountain slopes, forest edges, river banks.	On the steppe, gravelly and rocky mountain slopes.	In the foothills, along gravelly and loess slopes, rocky valleys.	On meadow mountain slopes, lawns and edges of walnut and deciduous forests.	On rocky scree, in pine forests, on dry elevated places on rocky and clay soil.
Distribution in Kazakhstan	Chu-Ili Mountains, Karatau.	Chu-Ili Mountains, Karatau, Western Tien Shan.	Tobolsk-Ishim, Irtysh, Semipalatinsk, Kokchetav, Caspian, Aktobe, Western and Eastern small hills, Altai.	Tobolsk-Ishim, Irtysh, Semipalatinsk, Kokchetav, Caspian, Aktobe, Altai, Turkestan, Dzungarian Alatau, Zaili Alatau, Chu-Ili, Karatau, Western Tien Shan.	Karatau.	Western Tien Shan.	Western Tien Shan.	Tobolsk-Ishim.

**Table 2:** Comparative data on extraction conditions and BAS content in some representatives of the *Salvia* genus common in Kazakhstan.

Species	Plant Part	Location	Extraction Method	Solvent	TPC (mg eq-QE/g)	TFC (mg eq-QE/g)	RA (mg/g)	LT (mg/g)	Ref.
*S. virgata*	L R S F	Iran	M	70% MeOH	35.6 ± 7.6 15.8 ± 3.3 18.7 ± 2.6 36.7 ± 5.7	83.9 ± 9.4 94.9 ± 4.7 82.1 ± 7.3 100.4 ± 8.7	ND	ND	[16]
	AP	Turkey	S	H EA MeOH 50% MeOH W	28.31 ± 0.58 64.47 ± 1.04 133.79 ± 0.79 212.30 ± 0.43 116.22 ± 0.84	0.81 ± 0.09 0.10 ± 0.02 6.53 ± 0.11 3.57 ± 0.05 3.87 ± 0.06	ND 4.48 ± 0.13 59.75 ± 1.66 48.49 ± 2.84 23.23 ± 0.43	ND 0.06 ± 0.00 0.14 ± 0.01 0.36 ± 0.02 0.58 ± 0.02	[17]
	AP	Turkey	M	80% MeOH	125.11 ± 5.44	28.03 ± 0.44	37.93 ± 1.42	ND	[18]
	AP	Turkey	M	70% MeOH W	195.22 ± 0.25 120.14 ± 2.27	62.20 ± 0.57 14.17 ± 0.83	66.94 ± 0.471 26.81 ± 0.047	0.97 0.22	[19]
	AP	Turkey	M	80% EtOH	283.35 ± 10.4	13.37 ± 1.6	ND	ND	[20]
	AP	Iran	M	MeOH	7.5 ± 0.7	ND	ND	ND	[21]
*S. macrosiphon*	L R	Iran	M	70% MeOH	14.7 ± 2.0 5.8 ± 0.8	51.9 ± 5.5 15.9 ± 1.9	ND	ND	[16]
	AP	Iran	M	80% MeOH	111.9 ± 0.06	ND	ND	ND	[22]
	AP	Iran	M	MeOH	7.1 ± 1.0	ND	ND	ND	[21]
	L	Iran	P	85% MeOH	ND	ND	ND	6.25%	[23]
*S. sclarea*	L R	Iran	M	70% MeOH	19.3 ± 2.3 1.5 ± 0.2	69.9 ± 3.2 14.9 ± 2.1	ND	ND	[16]
	L	Hungary	U	50% MeOH	3.07%	ND	0.87%	ND	[24]
	ND	Lithuania	SFE-CO_2_	EtOH	ND	ND	7.323 ± 0.084	ND	[25]
	AP	Moldova	M	EtOH	110.90 ± 0.26	ND	ND	ND	[26]
	AP	Iran	M	80% MeOH	14.13 ± 0.90	ND	ND	ND	[27]
	AP	Turkey	M U P	MeOH MeOH NADES	ND	ND	0.179 ± 0.00 0.191 ± 0.00 0.090 ± 0.00	0.009 ± 0.00 0.008 ± 0.00 0.002 ± 0.00	[28]
	WP	Ukraine	M	80% EtOH	29.39–91.02	25.91–53.82	–	–	[29]
	AP	Ukraine	U+M M P+M M	70% EtOH 65% EtOH 70% EtOH 70% EtOH	832.8 567.9 703.0 1045.9	1675.6 1142.01 1414.09 2103.12	ND	ND	[30]
	AP	Serbia	M U M U	MeOH MeOH 80% MeOH 80% MeOH	ND	ND	175.66 ± 2.02 177.77 ± 1.89 197.48 ± 2.00 171.99 ± 1.88	1.45 ± 0.01 1.13 ± 0.01 0.96 ± 0.02 0.80 ± 0.02	[31]
	AP	Kazakhstan	U	50% EtOH	ND	7.71%	ND	ND	[32]
*S. dumetorum*	ND	Lithuania	SFE-CO_2_	EtOH	ND	ND	5.024 ± 0.109	0.193 ± 0.094	[25]
	L	Hungary	U	50% MeOH	6.54%	ND	2.09%	ND	[24]
	L	Ukraine	U	MeOH	ND	ND	1.223	ND	[33]
*S. verticillata*	ND	Lithuania	SFE-CO_2_	EtOH W	ND	ND	15.436 ± 0.112 1.383 ± 0.032	0.294 ± 0.0075 0.402 ± 0.0057	[25]
	AP	Moldova	M	EtOH	107.62 ± 0.08	ND	ND	ND	[26]
	AP	Iran	U	80% MeOH	32.61 ± 0.39	11.32 ± 0.23	ND	ND	[34]
	L	Iran	M	MeOH	ND	ND	4.78	ND	[35]
	L	Greece	U	70% MeOH	ND	ND	223.12 ± 1.63	23.92 ± 5.7	[36]
	L	Turkey	S	MeOH	ND	ND	37.1	ND	[37]
	AP	Serbia	M	MeOH	175.6 ± 16.3	244.4 ± 4.7	23.458 ± 0.52	–	[38]
	L	Ukraine	U	MeOH	ND	ND	12.310	ND	[33]
*S. aethiopis*	AP	Moldova	M	EtOH	81.43 ± 0.25	ND	ND	ND	[26]
	AP	Iran	M	80% MeOH	14.13 ± 0.90	ND	ND	ND	[27]
	ND	Cyprus	M	MeOH	90.75	121.76	4.18 ± 0.9	5.56 ± 0.2	[39]
	ND	Turkey	M	EtOH	ND	ND	1.904	0.1715	[40]
	AP	Serbia	U	MeOH 80% MeOH EtOH 60% EtOH 80% EtOH EA	ND	ND	0.222 ± 0.030 0.223 ± 0.022 0.140 ± 3.21 0.0725 ± 0.00 0.231 ± 0.041 0.0058 ± 0.00	ND	[41]
	L	Ukraine	U	MeOH	ND	ND	0.552	ND	[33]
*S. deserta*	AP	Kazakhstan	U	50% EtOH	ND	10.14%	ND	ND	[32]
	L F	China	M	70% MeOH	ND	ND	30 8	ND	[42]
*S. trautvetteri*	No data available								

TPC—total phenolic content; TFC—total flavonoid content; RA—rosmarinic acid; LT—luteolin; L—leaves, R—roots, S—stem, F—flowers, AP—aerial parts, WP—whole parts; M—maceration, U—ultrasound, P—percolation, S—soxhlet, SFE-CO_2_—supercritical carbon dioxide extraction; NADES—natural deep eutectic solvent, MeOH—methanol, EtOH—ethanol, EA—ethyl acetate, H—hexane, W—water; ND—not determined.

**Table 3:** Phenolic compounds and terpenoids of *Salvia* species growing in Kazakhstan.

Number	Compound Name	Type	Reference
(1)	Caffeic acid	Hydroxycinnamic acid	[17,24,25,31,32,33,39,42,50]
(2)	Rosmarinic acid	Phenolic acids	[17,18,24,25,28,31,32,33,36,42,50]
(3)	Carnosic acid	Phenolic diterpene	[25,38]
(4)	Luteolin-7-glucuronide	Flavonoid	[25]
(5)	Apigenin-7-glucuronide	Flavonoid	[25]
(6)	Quercetin-3-glucuronide	Flavonoid	[25]
(7)	Gallic acid	Phenolic acid	[17,28,37]
(8)	p-OH-benzoic acid	Phenolic acid	[17]
(9)	o-coumaric acid	Hydroxycinnamic acid	[17,36,37]
(10)	Cynaroside	Flavonoid	[17,31,33]
(11)	Luteolin	Flavonoid	[17,28,31,32,36,39,40]
(12)	Rutin	Flavonoid	[18,28,33]
(13)	α-tocopherol	Tocopherol	[25]
(14)	γ-tocopherol	Tocopherol	[25]
(15)	δ-tocopherol	Tocopherol	[25]
(16)	Quercetin	Flavonoid	[28,33]
(17)	Catechin	Flavonoid	[21,28]
(18)	Syringic acid	Phenolic acid	[28]
(19)	Coumarin	Flavonoid	[28]
(20)	Myricetin	Flavonoid	[28]
(21)	Apigenin	Flavonoid	[25,28,31,32,33,36,40]
(22)	3-hydroxy benzoic acid	Phenolic acid	[28]
(23)	Ferulic acid	Hydroxycinnamic acid	[28,36]
(24)	Trans-cinnamic acid	Phenolic acid	[28]
(25)	Ellagic acid	Phenolic acid	[28]
(26)	Salvigenin	Flavonoid	[31,32,36]
(27)	Cosmosiin	Flavonoid	[31,33]
(28)	Cirsimaritin	Flavonoid	[32,33,36]
(29)	Kaempferol-3-*O*-glucoside	Flavonoid	[33]
(30)	6-hydroxyluteolin-5-glucoside	Flavonoid	[33]
(31)	Hispidulin	Flavonoid	[33,36]
(32)	Caftaric acid	Hydroxycinnamic acid	[36]
(33)	Dimer-β-(3,4-dihydroxyphenyl) lactic acid	Phenolic acid	[36]
(34)	Sagerinic acid	Cyclobutane lignans	[36]
(35)	Methyl-rosmarinic acid	Hydroxycinnamic acid	[36]
(36)	Salvianolic acid B	Stilbenoid	[32,36]
(37)	Salvianolic acid E	Stilbenoid	[36]
(38)	Salvianolic acid K	Stilbenoid	[36]
(39)	Lithospermic acid	Phenolic acid	[36]
(40)	Danshensu	Phenolic acid	[32,36]
(41)	Medioresinol	Furanoid ligans	[36]
(42)	p-coumaric acid	Hydroxycinnamic acid	[37,40]
(43)	Chlorogenic acid	Phenolic acid	[37,40]
(44)	Vanillic acid	Flavonoid	[32,37]
(45)	Carnosol	Phenolic diterpenoid	[38]
(46)	Salvianolic acid C	Stilbenoid	[38]
(47)	Isoquercetin	Flavonoid	[39]
(48)	Acecetin	Flavonoid	[39]
(49)	Fumaric acid	Dicarboxylic acid	[39]
(50)	Naringenin	Flavonoid	[39,40]
(51)	Quinic acid	Cyclohexanecarboxylic acid	[40]
(52)	Protocatechuic acid	Hydroxybenzoic acid	[40]
(53)	Hyperoside	Flavonoid	[40]
(54)	4-hydroxybenzoic acid	Hydroxybenzoic acid	[40]
(55)	Salicylic acid	Phenolic acid	[40]
(56)	Hesperetin	Flavonoid	[40]
(57)	Kaempferol	Flavonoid	[40]
(58)	Rhamnetin	Flavonoid	[40]
(59)	Chrystin	Flavonoid	[40]
(60)	Dihydrocaffeic acid	Hydroxycinnamic acid	[32]
(61)	Protocatechuicaldehyde	Hydroxybenzaldehydes	[32]
(62)	Luteolin 7-*O*-glucoside	Flavonoid	[32]
(63)	Apigenin 7-*O*-glucoside	Flavonoid	[32]
(64)	Tetramethoxeflavone	Flavonoid	[32]
(65)	Methoxycoumarin	Coumarin	[32]
(66)	Jaceosidin	Flavonoid	[32]
(67)	Viscosine	Flavonoid	[32]
(68)	Eupatorin	Flavonoid	[32]
(69)	Genkwanine	Flavonoid	[32]
(70)	Oleanolic acid	Triterpenoid	[50]
(71)	Ursolic acid	Triterpenoid	[50]
(72)	Salvisertin A	Triterpenoid	[51]
(73)	Dichroanone	Diterpenoid	[51]
(74)	Sugiol	Diterpenoid	[51]

**Table 4:** The content of VOCs identified in *S. virgata* essential oil ranged by country of origin and their relative percentages of the area (% area) according to the literature.

Component	Area, %								
	Iran				Turkey		Greece	Krym	Uzbekistan
	[16]	[52]	[53]	[54]	[55]	[56]	[57]	[58]	[59]
3-Ethyl-3-hydroxyandrostan-17-one	10.22	–	–	–	–	–	–	–	–
Aromadendrene	0.48	–	–	–	–	–	15.2	–	0.3
Bicyclogermacrene	–	–	–	1.85	–	–	0.6	–	–
Borneol	–	–	–	0.81	1.5	–	0.3	0.22	0.3
Camphene	–	–	–	–	–	–	2.2	3.19	2.6
Camphor	–	–	–	–	0.74	–	–	0.63	–
Carvacrol	–	–	–	–	–	–	–	–	0.5
Caryophyllene oxide	–	30.23	34.4	6.9	7.43	13.22	6.6	6.03	–
*cis*-Z-α-Bisabolene epoxide	2.73	–	–	–	–	–	–	–	–
*cis*-β-Faesene	1.48	–	–	0.78	–	–	0.5	–	–
Doconexent	3.97	–	–	–	–	–	–	–	–
Elemen	0.96	0.24	–	–	0.67	–	–	–	–
Eucalyptol	–	–	–	–	0.21	–	–	–	–
Falcarinol	2.07	–	–	–	–	–	–	–	–
Germacrene B	–	–	–	0.49	–	–	–	–	–
Germacrene D	–	–	–	3.23	6.01	9.75	6.1	0.34	–
Hexadecane	–	–	0.1	–	–	–	–	–	–
Humulene epoxide II	–	–	0.2	1.37	–	0.74	0.3	–	–
Limonene	–	1.45	–	–	–	–	0.8	–	6.2
Linalool	–	–	0.1	–	–	0.73	–	30.50	0.2
Linalyl acetate	–	–	–	–	–	0.64	–	22.05	0.2
Myrcene	–	–	–	–	–	-	0.8	0.50	–
Phytol	–	–	–	6.83	–	–	–	–	–
Sabinene	–	11.82	–		-	2.02	21.2	–	–
Selinene	0.48	–	–	0.51	–	–	–	–	–
Seychellene	1.20	–	–		–	–	–	–	–
Spathulenol	–	0.17	25.6	6.09	0.71	0.84	0.6	2.83	1.0
Squalene	–	–	–	0.86	–	–	–	–	–
Terpinolene	–	–	–	–	–	–	0.3	–	–
Thymol	0.34	0.75	0.2	-	–	–	–	–	0.3
*trans*-β-Ocimene	–	–	–	–	–	–	–	0.29	–
Valeranone	26.09	–	–	1.19	–	–	–	–	–
α-Pinene	–	1.14	–	–	–	–	1.9	–	–
α-Copaene	–	–	–	–	–	2.03	0.4	0.71	0.8
α-Gurjunene	5.62	-	–	–	–	–	–	–	–
α-Humulene	0.50	0.95	0.1	1.03	10.88	2.83	1.1	0.55	1.2
α-Terpineol	–	–	–	–	–	–	–	–	1.0
α-Thujone	–	–	–	–	0.2	0.82	–	0.12	–
β-Caryophyllene	7.08	22.6	3.1	1.96	36.26	48.12	–	–	–
β-Eudesmol	–	–	–	–	–	–	–	–	1.2
β-Bourbonene	–	–	–	–	1.82	–	–	0.19	–
β-Ionone	–	–	–	–	–	–	0.2	–	–
β-Pinene	–	–	–	0.28	1.05	–	1.3	–	–
γ-Terpinene	–	1.12	0.3	–	0.93	1.03	1.9	–	5.2
δ-Cadinene	23.3	–	–	0.44	0.15	3.6	0.3	–	–

**Table 5:** The content of VOCs identified in S. *macrosiphon* from Iran and their relative percentages of the area (% area) according to the literature.

Component	Reference					
	[16]	[22]	[60]	[61]	[62]	[63]
1,8-Cineole	–	–	5.82	–	1.1	–
4-Terpineol	–	–	0.16	–	1.0	–
Aromadendrene	2.88	–	–	1.1	–	0.39
Benzyl benzoate	–	–	–	–	–	0.68
Bicyclogermacrene	–	–	0.70	1.8	–	0.71
Borneol	0.77	–	–	–	–	–
Butyl benzoate	–	–	–	–	–	49.16
Camphene	–	–	0.18	–	–	–
Camphor	3.10	–	0.48	–	–	–
Carvacrol	–	9.96	–	–	–	–
Caryophyllene oxide	14.63	–	0.20	–	–	1.19
Cyperene	–	–	–	–	–	4.10
Ethyl benzoate	–	–	–	–	–	0.31
Fenchone	–	–	–	–	1.1	–
Germacrene A	–	0.91	–	–	–	–
Germacrene B	1.32	0.73	–	0.2	–	–
Germacrene D	7.59	0.93	2.14	4.3	–	1.62
Heptacosane	0.30	–	–	–	–	–
Hexanal	–	–	0.25	–	–	–
Hexanol	–	–	–	–	1.4	–
n-Hexyl benzoate	–	–	–	–	–	7.0
Hexyl isobutyrate	–	0.96	–	–	–	0.82
Hexyl isovaleriate	–	5.06	–	–	–	–
Hexyl n-valerate	–	–	–	4.8	–	–
Humulene epoxide II	0.22	–	–	–	–	–
Isocomene	–	1.15	–	–	–	–
Limonene	0.22	–	2.73	–	–	–
Linalool	27.20	19.00	0.34	–	54.8	3.31
Linalyl acetate	1.55	–	–	–	–	–
Manool	–	0.67	–	–	27.3	–
Manoyl oxide	–	–	–	–	1.3	–
Methyl benzoate	–	–	–	–	–	0.27
Metyl chavicol	–	–	–	–	1.1	–
Myrcene	–	–	1.01	–	–	–
o-Cymene	–	–	1.03	–	–	–
Octyl benzoate	–	–	–	–	–	2.75
p-Cymene	–	–	7.25	–	–	–
Sabinene	–	–	0.85	–	–	–
Sclareol	–	0.6	–	8.6	–	–
Spathulenol	5.86	–	–	5.80	1.7	4.83
Thymol	–	8.73	0.44	–	–	–
Valencene	–	–	–	1.4	–	–
α-Cadinene	–	1.04	–	1.8	–	–
α-Cubebene	–	–	0.38	–	–	–
α-Pinene	–	–	0.39	–	–	–
α-Caryophyllene	–	–	0.45	0.6	–	1.34
α-Copaene	–	–	1.26	0.9	–	–
α-Eudesmol	1.25	0.82	–	–	–	–
α-Gurjunene	–	–	–	0.6	–	–
α-Terpinene	–	–	1.11	2.6	–	–
α-Terpineol	0.21	–	–	–	1.7	–
α-Murolol	–	–	–	–	1.4	–
α-Thujene	–	–	4.28	–	–	–
α-Thujone	–	–	17.84	–	–	–
β-Caryophyllene	13.50	–	9.92	–	–	3.54
β-Cedrene	–	14.64	–	–	–	–
β-Cubebene	–	–	0.42	0.3	–	-
β-Elemene	–	13.33	–	5.4	–	3.02
β-Eudesmol	–	–	–	3.9	–	–
β-Fanesene	–	–	–	0.5	–	–
β-Selinene	–	3.18	0.40	2.2	–	–
β-Pinene	–	–	1.52	–	–	–
β-Thujone	–	–	1.83	–	–	–
γ-Elemene	–	0.6	0.39	0.2	–	–
γ-Terpinene	0.85	–	14.75	–	–	–
δ-Cadinene	–	–	1.32	0.7	–	–
δ-Elemene	–	4.02	–	–	–	–
δ-Selinene	–	–	–	–	–	2.86

**Table 6:** The content of VOCs identified in *S. sclarea* ranged by country of origin and their relative percentages of the area (% area) according to the literature.

Component	Area, %									
	Iran [16]	Uzbekistan [59]	Lithuania [64]	Poland [65]	Slovakia [66]	Tadjikistan [67]	Italy [68]	Bulgaria [69]	India [70]	Georgia [71]
1,8-Cineole	–	1.2	–	–	–	0.1	–	–	–	–
2-Tridecanone	–	0.8	1.2	–	–	–	–	–	–	–
9-octadecenoic acid	–	6.9	–	–	–	–	–	–	–	–
Aromadendrene	–	–	–	–	–	–	0.4	0.27	–	–
Bicyclogermacrene	–	–	–	–	0.2	1.2	0.8	–	–	–
Borneol	–	0.2	–	–	–	0.1	–	–	–	–
Camphene	–	–	–	23.36	–	–	–	–	–	–
Camphor	–	0.4	–	2.74	–	–	0.1	–	–	–
Carvacrol	–	–	4.0	–	–	1.3	–	–	–	–
Caryophyllene oxide	0.55	–	14.0	–	0.3	0.2	0.2	–	–	1.56
Elemen	–	–	–	–	–	0.2	–	–	–	–
Eugenol	–	1.1	–	–	–	–	–	–	–	–
Germacrene D	16.4	–	9.6	–	0.2	11.4	10.5	–	–	0.35
Hexacosane	–	–	6.6	–	–	–	–	–	–	–
Hexadecane	–	–	1.7	–	–	–	–	–	–	–
Humulene epoxide II	–	–	–	–	–	–	0.3	–	–	–
Limonene	–	0.4	–	–	2.20	0.2	0.1	1.05	–	–
Linalool	26.2	–	–	–	20.6	12.5	11.3	17.67	13.78	14.9
Linalyl acetate	20.5	4.7	–	–	49.1	39.2	59.3	34.62	20.58	18.8
Methyl eugenol	–	0.3	–	–	–	–	–	–	–	–
Myrcene	1.85	–	–	–	0.6	0.7	0.5	1.82	8.54	1.21
*n*-Butyloctadecenoate	–	5.7	–	–	–	–	–	–	–	–
Nerol	2	–	–	–	1.1	1.1	–	0.15	–	1.66
Selinene	–	–	–	–	–	0.2	–	–	–	–
Spathulenol	–	2.5	1.5	–	–	0.2	0.3	0.72	–	–
Squalene	–	–	10.9	–	–	–	–	–	–	9.07
Tetradecane	–	–	2.0	–	–	–	–	–	–	–
Thujol	–	–	–	12.31	–	–	–	–	–	–
Thymol	0.74	0.4	–	–	–	1.5	–	–	–	–
*trans*-β-Ocimene	1.82	0.5	0.7	–	0.5	0.2	0.1	1.59	–	0.5
Triacontane	–	–	1.3	–	–	–	–	–	–	–
α-Pinene	–	–	–	–	2.4	0.1	–	–	–	–
α-Caryophyllene	–	0.3	–	–	–	0.1	1.7	0.30	–	–
α-Copaene	–	1.3	4.9	–	0.2	1.0	1.2	–	–	–
α-Eudesmol	–	–	1.9	–	–	–	–	–	–	–
α-Terpineol	–	2.5	0.8	–	4.9	5.5	–	4.84	17.82	–
α-Thujone	3.21	–	–	–	–	0.4	–	–	–	–
β-Caryophyllene	–	–	–	–	5.1	2.4	3.7	5.6	27.00	1.23
β-Cubebene	6.18	–	12.3	–	–	–	–	–	–	–
β-Eudesmol	0.65	1.3	–	–	–	0.5	0.1	–	–	–
β-Bourbonene	0.71	–	–	–	–	–	–	0.38	–	–
β-Pinene	–	–	1.4	–	0.2	–	–	–	–	–
γ-Terpinene	–	0.3	2.2	–	–	0.3	–	–	–	–
δ-Cadinene	0.68	1.4	–	–	–	0.4	0.7	0.97	–	–

**Table 7:** The content of VOCs identified in *S. dumetorum* ranged by country of origin and their relative percentages of the area (% area) according to the literature.

Component	Area, %			
	Ukraine [33]	Lithuania [64]	Poland [65]	Kazakhstan [72]
1,8-Cineole	0.1	–	–	0.13
Aromadendrene	–	–	–	0.52
Bicyclogermacrene	–	–	–	5.59
Camphene	–	–	17.26	-
Camphor	–	–	2.66	0.89
Caryophyllene oxide	–	24.7	–	11.16
Docosane	0.9	–	–	–
Dotriacontane	3.6	–	–	–
Elemen	–	–	–	0.45
Germacrene D	–	–	–	1.13
Heneicosane	1.4	–	–	–
Hentriacosane	15.3	–	–	–
Heptacosane	2.4	–	–	–
Hexacosane	–	12.9	–	–
Hexadecane	–	–	–	0.22
Humulene epoxide II	–	1.0	–	1.07
Limonene	0.1	–	–	–
Linalool	–	–	–	0.14
Linalyl acetate	–	–	–	0.08
Nonacosane	5.3	–	–	–
Pentacosane	1.0	–	–	–
Phytol	–	6.7	–	–
Sabinene	–	0.6	–	–
Selinene	–	-	–	2.6
Spathulenol	–	4.8	–	10.75
Squalene	–	20.1	–	–
Tetradecane	3.0	1.0	–	0.16
Thujol	–	–	8.42	–
Thymol	–	–	–	0.46
Triacontane	–	9.0	–	–
Tritiacontane	12.9	–	–	–
α-Pinene	0.1	–	2.88	–
α-Humulene	–	–	–	1.82
α-Gurjunene	–	–	–	0.33
α-Ionol	–	0.9	–	–
α-Terpineol	–	–	–	0.20
α-Thujone	–	–	–	0.59
β-Caryophyllene	–	0.7	–	13.21
β-Cubebene	–	0.8	–	–
β-Famesene	–	–	–	0.18
β-Sitosterol	–	–	2.77	–
γ-Sitosterol	4.0	–	–	–
γ-Terpinene	–	–	–	0.11
δ-Cadinene	–	–	6.77	–

**Table 8:** The content of VOCs identified in *S. verticillata* ranged by country of origin and their relative percentages of the area (% area) according to the literature.

Component	Area, %							
	Greece * [57]	Lithuania [64]	Poland [65]	Italy [73]	Turkey [74]	Ukraine [75]	Serbia [76]	Iran [77]
1,8-Cineole	ND–18.4	–	–	–	2.5	6.757	–	–
4-Terpineol	ND–0.2	–	–	–	0.6	2.226	–	0.28
4aα,7α,7aα-Nepetalactone	ND–51.4	–	–	–	–	–	–	–
Aromadendrene	–	2.2	–	0.1–0.4	–	–	–	0.05
Bicyclogermacrene	ND–−0.1	–	–	11.5–14.8	–	6.631	–	6.32
Borneol	–	–	0.33	–	0.4	–	–	0.20
Camphene	–	–	2.39	–	–	0.799	–	0.18
Camphor	ND–0.1	2.8	5.23	–	0.6	2.757	–	–
Carvacrol	–	2.5	–	–	2.1	–	–	–
Caryophyllene oxide	2.6–6.0	–	–	1.0	1.9	4.355	–	0.89
*cis*-Z-α-Bisabolene epoxide	–	–	–	–	–	–	–	0.58
Eucaliptol	–	–	0.80	–	–	–	–	–
Germacrene B	–	35.7	–	–	–	–	–	–
Germacrene D	11.5–2.8	14.3	-	39.5–40.7	–	11.881	–	0.21
Hecadecanoic acid	–	–	–	–	2.3	-	–	-
Hexacosane	–	0.8	–	–	–	–	–	–
Hexadecane	–	2.0	–	–	–	–	–	–
Hexanal	–	–	–	–	0.5	–	–	–
Humulene epoxide II	0.4–1.0	–		0.2–0.4	0.9	–	–	–
Limonene	1.8–ND	–	5.85	3.9	4.1	–	–	3.80
Linalool	0.2–0.1	–	–	0.4–0.8	2.2	0.310	–	0.05
Myrcene	0.3–0.2	–	–	0.8–1.1	1.4	0.142	6.0–6.6	1.92
Myrtenol	ND–0.1	–	–	–	–	–	–	–
Nerol	–	28.1	–	–	–	–	–	–
Nerolidol	35.0–ND	–	–	–	–	–	–	–
o-Cymene	–	–	–	–	–	–	0.5–0.6	–
p-Cymene	–	–	–	0.2–0.3	1.0	–	–	0.22
Phytol	–	–	–	–	–	–	–	0.84
Sabinene	0.2–0.6	–	–	0.8	–	–	1.7–5.5	4.44
Sabinene hydrate	ND–0.1	–	–	–	–	–	–	–
Seychellene	–	13.1	–	–	–	–	–	–
Spathulenol	1.1–0.1	44.2	–	3.1–6.6	31.0	–	-	5.89
Squalene	–	0.7	–	–	–	–	–	–
Tetradecane	0.3–ND	–	–	0.4–−0.7	–	–	–	–
Thymol	–	–	–	–	0.4	–	–	–
Thujol	–		2.18	–	–	–	–	–
Cis-ocimene	–		1.54	–	–	–	–	–
*trans*-β-Ocimene	–	7.0	–	0.6–2.9	–	–	–	1.65
Tridecsne	–	–	–	0.4–0.3	–	–	–	–
Valeranone	–	–	–	–	–	3.298	–	0.38
Verbenone	–	–	–	–	0.8	–	–	–
Virodoflorol	ND–0.2	–	–	–	–	15.502	–	0.12
α-Pinene	1.0–0.6	–	10.72	0.5–1.3	8.2	0.145	1.9–21.1	3.03
α-Copaene	2.3–0.2	–	–	0.3–0.5	–	–	–	0.25
α-Cubebene	0.4–ND	–	–	0.7–0.1	–	–	–	0.23
α-Gurjunene	0.4–ND	2.5	–	–	–	–	–	3.22
α-Humulene	0.7–0.4	–	–	2.7–5.9	–	5.864	0.2	8.61
α-Terpineol	ND–1.2	–	–	–	1.0	–	–	–
α-Thujone	–	–	–	0.3	–	11.013	1.6–2.5	–
β-Phellandrene	–	–	–	4.5–4.9	–	–	43.9–55.5	9.08
β-Caryophyllene	3.8–1.3	5.7	–	7.3–11.9	0.7	–	0.3–0.9	24.40
β-Copaene	ND–0.1	–	–	0.6–0.7	–	–	–	–
β-Cubebene	0.8–ND	–	–	0.3	–	–	–	0.23
β-Elemene	0.7–0.2	–	–	0.3–0.4	–	–	–	–
β-Farnesene	–	–	–	1.5–2.4	–	–	–	–
β-Bourbonene	3.0–1.3	–	–	1.8–3.1	–	0.368	–	0.17
β-Pinene	6.1–1.6	–	2.49	2.6–3.7	2.0	–	3.0–3.6	5.00
β-Thujone	–	–	–	–	–	0.932	–	–
γ-Cadinene	0.8–ND	–	–	1.5–0.3	–	–	–	–
γ-Muurolene	1.2–ND	–	–	0.2–0.4	–	–	7.9	–
γ-Terpinene	ND–0.7	–	–	–	1.1	–	–	0.21
δ-Cadinene	1.2–0.2	–	–	0.7–1.2	–	0.302	–	–
δ-Elemene	–	–	–	1.4–7.4	–	–	–	–

* Data on *S. verticilliata* from Greece present VOCs in cultivated and wild-growing plants (cultivated–wild growing).

**Table 9:** The content of VOCs identified in *S. aethiopis* ranged by country of origin and their relative percentages of the area (% area) according to the literature.

Component	Area, %			
	Greece [57]	Serbia [76]	Turkey [78]	Bulgaria * [79]
Bicycloelemene	–	–	1.27	–
Bicyclogermacrene	0.6	–	2.55	–
Camphene	0.1	–	–	–
Caryophyllene oxide	8.4	–	1.82	0.09–0.35
Germacrene A	0.5	–	–	–
Germacrene D	20.0	–	15.20	29.37–21.19
Heptacosane	–	–	–	0.69–0.20
Hexacosane	–	–	–	1.73–0.51
Humulene epoxide II	1.3	–	–	–
Phytol	0.6	–	–	–
Sabinene	0.3	0.8	–	–
Spathulenol	–	–	–	0.10–0.24
Valeranone	0.5	–	–	–
Viridiflorol	–	–	0.80	–
α-Pinene	0.3	1.3	–	0.17–0.19
α-Copaene	13.7	33.4	16.46	13.55–17.24
α-Cubebene	0.4	–	0.81	0.97–0.64
α-Humulene	6.4	1.5	7.4	5.46–6.79
α-Muurolene	–	7.2	–	–
β-Caryophyllene	30.6	36.8	30.46	23.55–21.91
β-Cubebene	4.4	–	7.04	7.02–9.71
β-Elemene	1.1	7.3	3.01	–
β-Bourbonene	0.7	0.2	–	–
β-Pinene	0.2	0.7	–	0.20–0.23
γ-Cadinene	–	–	–	0.98–0.63
γ-Elemene	–	–	–	0.62–1.28
γ-Muurolene	–	10.3	–	–
δ-Cadinene	3.6	0.2	5.73	5.56–6.69

* Data on *S. aethiopis* from Bulgaria present VOCs in flowers and leaves (flowers–leaves).

**Table 10:** The content of VOCs identified in *S. deserta* ranged by country of origin and their relative percentages of the area (% area) according to the literature.

Component	Area, %	
	China * [42]	Poland [65]
1-Octen-3-ol	4.98–ND	–
4-Terpineol	ND–10.91	–
Camphene	–	4.01
Caryophyllene oxide	5.99–4.95	–
Eucalyptol	ND–0.73	–
Germacrene D	ND–0.94	–
Humulene epoxide II	0.94–1.26	–
Ledol	8.36–6.98	–
Limonene	ND–0.96	7.45
Manool	1.39–4.83	–
Myrcene	ND–0.71	–
o-Cymene	ND–2.15	–
Thujol	–	2.79
α-Pinene	ND–0.68	35.35
α-Copaene	ND–0.84	–
α-Humulene	ND–0.99	–
α-Terpineol	ND–0.72	–
β-Caryophyllene	0.25–4.6	–
β-Phellandrene	ND–29.74	–
β-Pinene	–	13.02
β-Terpineol	ND–2.62	–
γ-Muurolene	0.21–0.16	–
γ-Terpinene	ND–3.02	–
δ-Cadinene	ND–0.33	–

* Data on *S. deserta* from China present VOCs in leaves and flowers (leaves–flowers).

**Table 11:** Biological activity of eight *Salvia* species plants from Kazakhstan.

Species	Biological Activity	Assay Results	Ref.
*S. virgata*	Lipid peroxidation	Methanol, water, ethyl acetate, and hexane extracts inhibited β-carotene/linoleic acid co-oxidation in the range of 40%.	[17]
	Antioxidant	The IC_50_ for DPPH for the fractions: aqueous methanol 0.2 mg/mL > water 0.3 mg/mL > methanol 0.4 mg/mL > ethyl acetate 1.65 mg/mL.	[17]
		Non-digested methanolic extract IC_50_ for DPPH 0.57 ± 0.57 μg/mL, FRAP 1.43 ± 0.18 mM FeSO_4_ eq.in 1 g sample.	[18]
		FRAP value was 27.5 ± 1.2 μM eq-QE/g dry weight and IC_50_ DDPH scavenging 644.8 ± 65.3 μg dry weight/mL.	[21]
		The IC_50_ of EO for DPPH: 1.98 ± 0.23 mg/mL, in ABTS: 0.75 ± 0.02 mg/mL, in CUPRAC: 0.39 ± 0.02 mg/mL, in FRAP: 0.28 ± 0.01 mg/mL.	[59]
		Ethanolic extract IC_50_ for DPPH 291.58 ± 0.004 μg/mL, IC_50_ values for ABTS 16.74 ± 0.007 μg/mL.	[20]
		The IC_50_ of EO for DPPH: 22.12–24.45 mg/mL, for FRAP 26.84–28.46 μM eq-QE/g dw.	[81]
	Enzyme inhibitory	Non-digested methanolic extract showed α-glucosidase, α-amylase, AChE, and BChE inhibition activity in a concentration of 1 mg/mL was 70.2, 81.2, 16.45, and 25.98%; in a concentration of 0.5 mg/mL it was 55.7, 64.4, 11.87, and 17.85%, respectively. AGEs inhibitory potential for 1 mg/mL was 86.2%, and for 0.5 mg/mL, it was 55.5%.	[18]
		EO showed high BChE activity IC_50_ of 0.60 ± 0.01 mg/mL. While EO did not exhibit any α-glucosidase inhibition and AChE, it also exhibited weak activity as an α-amylase inhibitor.	[59]
		Ethanolic extract α-glucosidase and α-amylase inhibition activity were 75.73 and 62.72%, respectively.	[20]
	Cytotoxic	Ethanolic extract decreased MDA-MB-231 cell viability (*p* < 0.05) in a dose-dependent manner (IC_50_ = 0.118 mg/mL), and displayed no considerable cytotoxicity on the L929 cell line (0.0625–1 mg/mL).	[20]
	Antimicrobial	EO has the high activity against *Staphylococcus epidermidis*, *Penicillium funiculosum*, and *Escherihia coli.*	[52]
		Ethanolic extract has moderate activity towards *E. coli* (0.312 mg/mL) and *Staphylococcus aureus* (0.312 mg/mL), and weak antimicrobial activity towards *Bacillus cereus* (0.625 mg/mL).	[20]
		EO showed moderate antimicrobial activity against *S. aureus* and *Candida albicans.*	[81]
*S. macrosiphon*	Antioxidant	The highest radical scavenging activity was in roots, IC_50_ = 10.9 μg/mL, followed by leaves, IC_50_ = 36.7 μg/mL.	[16]
		FRAP value was 17.4 ± 2.3 μM eq-QE/g dry weight and IC_50_ DDPH scavenging was 415.3 ± 17.9 μg dry weight/mL.	[21]
		The IC_50_ determined using DDPH of EO and the total extract was 1.83 μg/mL and 55.07 μg/mL, respectively.	[22]
		Methanolic extract possesses activity FRAP 404.12 mmol of FeSO_4_/100 g dried plant.	[82]
		The IC_50_ DDPH of methanolic extract of aerial part showed higher activity than ethanolic and *n*-hexane extracts quantified as 230.29 ± 0.64 mg/mL.	[83]
		DPPH scavenging potential (78.0 ± 2.0%) of stem methanolic extract.	[84]
	Cytotoxic	The EO revealed a potent cytotoxic activity on MCF-7, MDA-MB-231, and T47D cell lines (IC_50_ < 0.15 μg/mL). Total extract exhibited moderate cytotoxic activity (IC_50_ < 100 μg/mL).	[22]
		Significant cytotoxicity of *n*-hexane fraction from hydromethanolic extract against cancerous cell lines A549, MCF-7, and MDA-MB-231 with IC_50_ 20.89 ± 0.35, 10.24 ± 0.15, and 20.98 ± 0.25 μg/mL, respectively.	[85]
	Enzyme inhibitory	AChE IC_50_ = 0.169 ± 0.045, while BChE activity was >0.5 μg/mL. Total extract did not exhibit AChE and BChE inhibitory effects at concentrations up to 500 μg/mL.	[22]
	Antimicrobial	MIC and MBC against *S. aureus* were 2.5 and 5 mg/mL, *Listeria monocetogenes* 2 and 10 mg/mL, *Salmonella enterica* 5 and 10 mg/mL, and there was no activity against *B. cereus*.	[62]
		*n*-hexane fractions from hydromethanolic extract MIC against *S. aureus* and *E. coli* were 1.25 and 2.50 mg/mL.	[85]
		The MIC value of methanolic extract of aerial part against *S. aureus* was 1.25 mg/mL, *S. epidermidis* 2.5 mg/mL, *Bacillus subtilis* 2.5 mg/mL, *K. pneumonia* 5 mg/mL, and *Pseudomonas aeruginosa* 10 mg/mL. The MIC value of ethanolic extract of aerial part against *S. aureus* was 2.5 mg/mL, *S. epidermis* 2.5 mg/mL, *B. subtilis* 2.5 mg/mL, *K. pneumonia* 10 mg/mL, and *P. aeruginosa* 10 mg/mL. The MIC of *n*-hexane extract of aerial part against *S. aureus* was 5 mg/mL, *S. epidermidis* 5 mg/mL, and *B. subtilis* 5 mg/mL.	[83]
		Stem butanol extract has activity against *P. aeruginosa* with a ZOI = 23 ± 2.00 mm.	[84]
	Antifungal	The MICs of EO against *C. albicans*, *Candida parapsilosis*, and *Candida glabrata* were 0.44, 0.056, and 0.088 μL/mL.	[63]
		Stem methanol extract showed the ZOI (11 ± 0.67 mm) against *Fusarium brachygibbosum*.	[84]
	Anti-inflammatory	It was revealed that only the highest examined dose of the seed EO was somehow effective in the early phase of the formalin test in rats.	[86]
	Analgesic	Seed EO could not significantly inhibit the neutrophil-induced damage by reducing MPO activity in the paws of the rat.	[86]
	Antidepressant	The results did not show any significant effect of hydroethanolic extract on immobility, swimming, and climbing behaviors. However, aqueous extract decreased immobility at doses of 300, 600, and 900 mg/kg (11.1, 13.3, and 14.2%, respectively, *p* < 0.05), and increased swimming at doses of 600 and 900 mg/kg (53.3% and 61.8%, *p* < 0.05) as compared with the control group.	[87]
	Locomotors	Significant decreases in locomotor activity were found in the hydroethanolic extract at doses of 1800 mg/kg.	[87]
*S. sclarea*	Antioxidant	The IC50 for root extract in DPPH assay was 14.9 μg/mL.	[16]
		The IC50 DPPH of methanolic extract 190.74 ± 5.7 μg plant extracted or μg eq-QE/1 mL 10^−4^ M DPPH.	[26]
		The IC_50_ for extract in the DPPH test was 32.33 ± 0.35, ABTS 17.20 ± 0.10, 29.67 ± 0.02 μg/mL.	[27]
		The green and methanolic extracts exhibited potent LPO activity with IC_50_ 5.61 ± 0.47 and 5.37 ± 0.27; CUPRAC A_0.5_ = 12.28 ± 0.12 and A_0.5_ = 17.22 ± 0.36; DDPH with IC_50_ = 6.31 ± 0.23 and IC_50_ = 19.20 ± 0.70; and ABTS+ with IC_50_ = 6.50 ± 0.45 and 8.64 ± 0.63 μg/mL, respectively.	[28]
		Extract had significantly high antioxidant activity in MDA tests 521.5 ± 16.2 pmol/mg, while in DPPH and FRAP tests it had lower activity than vitamin C and Trolox 29.52 ± 4.7%, FRAP 26.23 ± 3.8 mg-trolox/g, respectively.	[39]
		The antioxidant activity of EO is lower compared to the Trolox. DPPH radical scavenging ability was determined at 11.76 ± 1.34% inhibition, and the ABTS radical cation at 29.70 ± 1.45%.	[66]
		The IC50 for EO in the DPPH assay was 123 ± 0.99 μg/mL.	[68]
		The DDPH radical scavenging activity of methanolic extract in a concentration of 10 mg/mL was higher than ethanolic extract measured as 88.49 ± 2.63 and 82.21 ± 1.79%.	[88]
	Antimicrobial	The methanolic extract MIC against *E. coli* was 5 mg/mL, *Klebsiella pneumoniae* 2.5 mg/mL, *Salmonella* Typhi 0.31 mg/mL, *B. subtilis* 5 mg/mL, *S. epidermidis* 0.31 mg/mL, and *S. aureus* 1.25 mg/mL.	[26]
		The extract indicated moderate activity against *S. aureus* and *Streptococcus pneumoniae* at 1.25 mg/mL.	[27]
		The methanolic extract MIC = 0.5 mg/mL showed high activity against *E. coli.* The ultrasonic extract and green extract MICs against *E. faecalis* were 0.625 and 1.25 mg/mL, and against *P. aeruginosa* (strain PA01) were 2.5 and 1.25 g/mL, respectively. The green extract showed the best effect against *S. aureus*, MIC = 0.625 mg/mL. The MIC values of methanolic, ultrasonic, and green extracts against *Chromobacterium violaceum* (strain CV026) were recorded as 0.5, 0.25, and 0.25 mg/mL, respectively.	[28]
		Hydromethanolic extract had strong effect on *P. aeruginosa* with an MIC and MBC of 50 mg/mL. Methanolic extract prepared by ultrasound showed the best antimicrobial activity toward *S. aureus*, *B. cereus*, *Listeria monocytogenes*, *P. aeruginosa*, and *Enterobacter aerogenes* with the MIC values of 6.25, 12.5, 25, 50, and 50 mg/mL, respectively.	[31]
		The EO indicated high antimicrobial activity against *S. aureus*, MIC_50_ = 1.48 μL/mL, and biofilm-forming *Pseudomonas fluorescens*, MIC_50_ = 2.93 μL/mL.	[66]
		EO has activity against *S. aureus*, *P. aeruginosa*, *L. monocytogenes*, and *S. enterica* with MIC values of 5.6 ± 0.68, 11.2 ± 0.31, 4.6 ± 0.31, and 7.5 ± 0.0 mg/mL. In addition, EO showed the same MIC value of 7.5 mg/mL for *B. cereus*, *Enterococcus faecalis*, and *Micrococcus luteus*.	[68]
	Cytotoxic	A slight increase was found in the number of viable cells at tested doses for the extract on MCF-7 and MDA-MB-231 cell lines.	[27]
		The cytotoxic effects of conventional and ultrasonic extracts on HDFn using the MTT assay do not cause a significant toxic effect on human fibroblasts.	[32]
	Enzyme inhibitory	The green extract showed a maximum AChE inhibition of 47.00 ± 1.50% with IC_50_ 200 μg/mL, and BChE inhibition of 61.79 ± 0.63% with IC_50_ 131.6 ± 0.98 μg/mL. The methanolic extract inhibited 50.70 ± 0.94% BChE with IC_50_ 192.4 ± 1.25 μg/mL. The ultrasonic and methanolic extracts have moderated urease inhibitory activity of 55.12 ± 0.88% and 52.31 ± 0.74% with an IC_50_ of 171.6 ± 0.95 μg/mL and 187.5 ± 1.32 μg/mL, respectively.	[28]
		The ethanolic extract inhibitory effect on AChE was nearly four times higher with an IC_50_ of 0.27 ± 0.005 mg/mL, while the methanolic extract effect on MAO-A was found to be higher, with an IC_50_ of 3.03 ± 0.05 mg/mL.	[88]
	Spasmolytic	The hydromethanolic extract prepared by maceration was reported as the strongest bronchodilator agent, which inhibited spontaneous ileal contractions, and carbachol- and KCl-induced tracheal smooth muscle contractions. Methanolic extract by maceration was indicated as the causing most powerful relaxation of KCl-induced ileal contractions. Hydromethanolic extract by ultrasound generated the best spasmolytic effects in the acetylcholine-induced ileal contractions.	[31]
	Immunomodulatory	Immunomodulatory effects of the conventional and ultrasonic extracts had either a stimulating effect on inactivated macrophages or suppressed cytokine-producing activity in LPS-activated macrophages. Conventional extraction significantly increased the secretion of TNF-α by intact and Con A-activated splenic T lymphocytes.	[32]
	Anti-inflammatory	The IC50 of extracts was significantly lower compared to the anti-inflammatory IC50 of acetylsalicylic acid. IC_50_ for heat-induced hemolysis was 3.96 ± 0.86 mg/mL, for proteinase was 3.75 ± 0.71 mg/mL, and for albumin denaturation was 4.69 ± 0.96 mg/mL.	[39]
		The EO at concentration 80 μg/mL exhibited a 74% inhibition of nitric oxide with an IC_50_ of 37 ± 3 μg/mL.	[71]
	Antifungal	The best effect of EO against *C. tropicalis* was an MIC_50_ of 2.93 μL/mL. The ZOI against *Aspergillus flavus* at a concentration of 500 μL/mL was (8.00 ± 3.00 mm), *Botrytis cinerea* at a concentration of 250 μL/mL was (9.67 ± 1.53 mm), and *Penicillium citrinum* at a concentration of 250 μL/mL was (7.33 ± 0.58).	[66]
	Insecticidal	EO showed insecticidal activity on *Oxycarenus lavaterae*. The best activity was found for a 100% concentration of EO.	[66]
	Anti-trypanosomal	Water, methanolic, chlorophorm, and *n*-hexane extracts have activity against protozoan *Trypanosoma brucei rhodesiense* with an IC_50_ of 10.31, 6.44, 4.4, and 2.4 μg/mL, and for *Trypanosoma cruzi*, >90, 56.82, 52.51, and 18.17 μg/mL, respectively.	[89]
	Anti-leishmanial	Water, methanolic, chlorophorm, and *n*-hexane extracts have activity against protozoan *Leishmania* spp. with an IC_50_ of 47.88, 12.95, 8.31, and 5.25 μg/mL, respectively.	[89]
	Anti-plasmodial	Water, methanolic, chlorophorm, and *n*-hexane extracts have activity against protozoan *Plasmodium falciparum* with an IC_50_ of >20, 6.6, 2.54, and 3.78 μg/mL, respectively.	[89]
*S. dumetorum*	Cytotoxic	Hexane extract at a concentration of 10 μg/mL showed cell proliferation on the MCF-7 cancer cell line of 27.8 ± 1.0%, while chloroform extract has activity on the A431 cell line of 41.1 ± 0.9%.	[90]
	Antimicrobial	A 30% flower ultrasonic extract has the most pronounced activity towards *S. aureus* with a ZOI of 35 ± 1 mm, 40% leaf ultrasonic extract towards *Bacillus subtilis* with a ZOI of 49 ± 1 mm, and 70% leaf ultrasonic extract towards *E. coli* with a ZOI of 24 ± 1 mm.	[91]
*S. verticillata*	Antioxidant	The IC_50_ for extract in the DPPH test was 27.36 ± 0.32 μg/mL, ABTS 13.40 ± 0.10 μg/mL, 19.75 ± 0.02 μg/mL.	[27]
		Methanolic extract has antioxidant capabilities in the DPPH (58.05 mg AAE/g DW) and FRAP (41.38 μmol Fe^++^/g DW) assays.	[34]
		The scavenging potential of the hydromethanolic extract towards the DPPH free radical (IC_50_ = 15.8 μg/mL) was higher than the BHT (IC_50_ = 94.6 μg/mL).	[36]
		The antioxidant potential of methanolic and dichlormethane extracts in the DPPH assay was 16.0 ± 2.12 and 21.3 ± 0.86 mg/mL, and in the β-carotene/linoleic acid assay was 61.3 ± 5.38 and 30.4 ± 1.21 mg/mL.	[37]
		The scavenging potential of methanolic extract for the DPPH assay was IC_50_ 33.04 ± 5.83 μg/mL, ABTS^+^ assay was IC_50_ 67.01 ± 13.62 μg/mL, NO radical scavenging assay was IC_50_ 73.12 ± 19.04 μg/mL, and MDA assay was 58.07 ± 9.72 μg/mL.	[38]
		Five assays were used to determine the antioxidant capacity of the chloroform and petroleum ether extracts. Chloroform extract exhibited higher values in the FRAP and CUPRAC assays, measured as 50.22 ± 0.65 μgTE/mg and 69.90 ± 0.36 μgTE/mg, than petroleum ether extract, 30.12 ± 0.45 μgFE/mg and 38.58 ± 0.59 μgFE/mg. Results in ABTS, DPPH, and TRP fluorometric assays correlated with each other.	[92]
		Leaf ethanolic extract showed a high potency of free radical scavenging in DPPH assay with an IC_50_ value of 2.49 µg/mL and demonstrated the strongest antioxidative properties equal to Trolox (IC_50_ = 2.50 µg/mL). The ethanolic extract of leaves possesses NO radical scavenging activity at concentrations of 100, 200, and 400 µg/mL with IC_50_ 65.04 + 3.13, 80.17 + 3.46, and 81.55 + 1.72 µg/mL, respectively.	[93]
	Antimicrobial	The MIC values of the extract were 1.25 mg/mL against *S. aureus* and 2.5 mg/mL against *S. pneumoniae*, *E. coli*, *P. aeruginosa*, and *C. albicans.*	[27]
		Methanolic extract was very active on *B. cereus* with growth inhibition at a concentration of 1.25 mg/mL.	[38]
		Petroleum ether had activity against *Salmonella Enteritidis*, *E. coli*, *E. aerogenes*, *Enterococcus faecalis*, and *S. aureus* with an MIC value of 6.25 mg/mL, while chloroform extract had the same MIC value against *B. cereus.*	[92]
	Cytotoxic	A slight increase was found in the number of viable cells at tested doses for the extract on MCF-7 and MDA-MB-231 cell lines.	[27]
		The *S. verticillata* extract was tested at concentrations of 10, 25, and 50 μg/mL. High concentrations of the extract at 50 μg/mL exhibit significant cytotoxicity in a dose-dependent manner on the CBPI compared to the control. MMC cells treated with extract exhibit cytotoxicity in all concentrations.	[36]
		EO has demonstrated cytotoxic activity in HT-29, T-47D, Caco-2, and NIH-3T3 cell lines with IC_50_ 90.90 ± 14.88, 80.20 ± 8.91, 125.12 ± 27.59, and 81.81 ± 3.47 μg/mL, respectively.	[77]
		Chloroform extract at a concentration of 10 μg/mL showed cell proliferation on MCF7 and A431 cancer cell lines of 30.9 ± 0.6 and 48.3 ± 1.5%, while hexane extract has activity only on the A431 cell line at 32.1 ± 0.6%.	[90]
		The IC_50_ value for chloroform extracts was on MDA-MB-231 77.16 μg/mL and HCT 116 105.08 μg/mL cell lines; for petroleum ether extract, the IC_50_ value was 30.90 μg/mL for the MDA-MB-231 cell line and 44.28 μg/mL for the HCT 116 cell line.	[92]
		The methanolic extract of leaves was studied in an MTT assay on human cancer cell lines (MCF-7, SH-SY5Y, and HL-60) with an IC_50_ of 166.3 ± 2.4, 72.8 ± 1.9, and 127.8 ± 2.7 μg/mL, respectively.	[94]
	Genotoxic and antigenotoxic	Cells treated with hydromethanolic extract at concentrations of 10, 25, and 50 μg/mL showed low MN frequencies compared to control, thus indicating the absence of genotoxicity.	[36]
	Antifungal	The lowest MIC value of methanolic extract was found on *Penicillium canescens* (5 mg/mL), *C. albicans*, and *Fusarium oxysporum* (10 and 20 mg/mL, respectively).	[38]
		The MIC of the leaf, rootstock, and the combined ethanol extracts ranged from 3.12 to 25, 6.25 to 25, and 1.56 to 12.5 mg/mL, respectively. The combined extracts have shown strong antifungal effect against *Cryptococcus laurentii*, *Cryptococcus neoformans*, and *Geotrichum capitatum* with MIC values of 1.56 mg/mL, followed by *C. glabrata* with an MIC value of 3.12 mg/mL, and *C. albicans* and *Candida guillermondii* with MIC values of 6.25 mg/mL. *Candida tropicalis*, *C. parapsilosis*, *Debaryomyces hansenii*, and *Kluyveromyces fragilis* have shown a moderate activity with an MIC value of 12.5 mg/mL.	[95]
	Enzyme inhibitory	The inhibition of the AChE of ethanolic leaf extract with an IC_50_ of 1600 μg/mL was 51.30%, while less concentrations were inactive.	[93]
*S. aethiopis*	Antioxidant	The IC50 DPPH of methanolic extract was 123.37 ± 8.05 μg plant extracted or μg eq-QE/1 mL 10^−4^ M DPPH.	[26]
		The IC_50_ for extract in the DPPH test was 146.6 ± 1.1 μg/mL, ABTS 59.16 ± 0.05 μg/mL, 80.02 ± 0.05 μg/mL.	[27]
		MDA values of 521.5 ± 16.2 pmol/mg of the extract were found to be significantly higher compared to reference vitamin C 105.2 ± 15.8 pmol/mg. The DPPH scavenger and FRAP activities had significantly lower levels for the extract, quantified as 29.52 ± 4.7% and 26.23 ± 3.8 mg-trolox/g, respectively.	[39]
		In the CUPRAC assay, the water extract showed an activity similar to ascorbic acid. In the FRAP assay, the ethanol extract showed higher reducing power at a concentration of 30 μg/mL, the same as BHT. In the DPPH assay, it showed low antioxidant activity.	[40]
		The 80% methanolic extract was the most efficient in the DPPH antioxidant assay with IC_50_ 23.79 ± 0.42 μg/mL, while 60% ethanolic extract expressed the best anti-lipoperoxidant activity in the β-carotene/linoleic acid assay at 37.82 ± 1.45 μg/mL.	[41]
	Antimicrobial	The methanolic extract has an MIC against *Salmonella* Typhi of 5 mg/mL and against *K. pneumoniae* of 10 mg/mL.	[26]
		The extract indicated activity against *S. aureus*, *P. aeruginosa*, and *S. pneumoniae* with MIC values of 2.5 mg/mL and against *E. coli* and *C. albicans* with MIC values of 5 mg/mL.	[27]
		ZOIs of the extract for *S. aureus*, *E. aerogenes*, *E. coli*, and *P. aeruginosa* were 12 ± 0.00, 10 ± 0.47, 10 ± 0.00, and 9 ± 0.47 mm, respectively.	[40]
		The EO MIC assessed for the *Staphylococcus* strain was at 0.25% oil concentration.	[96]
	Cytotoxic	It was found that there was a slight increase in the number of viable cells at tested doses for the extract on MCF-7 and MDA-MB-231 cell lines.	[27]
		Hexane extract at a concentration of 10 μg/mL showed cell proliferation on MCF7 and A431 cancer cell lines of 33.5 ± 1.5 and 20.3 ± 1.3%.	[90]
	Anti-inflammatory	In heat-induced hemolysis, proteinase inhibitory activity, and albumin denaturation in vitro assays, low anti-inflammatory activity was found compared to the IC_50_ value of a standard drug.	[39]
	Inhibition of cannabinoid and opioid receptors	The ethanolic extract of aerial parts showed a moderate level of inhibition in cannabinoid (CB1—37.0% displacement and CB2—31.0% displacement) and opioid (Delta—46.3%, Kappa—45.3%, and Mu—32.9% displacement) receptors.	[80]
	Enzyme inhibitory	The EO inhibited 46.4 ± 0.8% (*n* = 3) of AChE activity, at 1 mg/mL.	[97]
*S. deserta*	Cytotoxic	The cytotoxic effects of conventional and ultrasonic extracts on HDFn using the MTT assay do not cause a significant toxic effect on human fibroblasts.	[32]
	Antithrombotic	Ethanolic root extract had significant inhibitory effects on ADP-induced maximum platelet aggregation rate (10.2 ± 2.6 vs. control 35.7 ± 5.2), reduced the FeCl_3_-induced rat common carotid artery thrombus weight and thrombus area ratio, significantly decreased plasma TXB_2_, vWF, and PAI-1 levels and increased 6-keto-PGF_1α_ and t-PA levels in a dose-dependent manner. Thus, the ratio of TXB_2_/6-keto-PGF_1α_ was significantly decreased, while the ratio of t-PA/*P*AI-1 was significantly increased. Enhanced AT-III and PC activities indicated coagulation inactivation effects of ethanolic root extracts.	[98]
	Anti-plasmodial	Diterpenoids from the root extract possessed weak activity with values of 1.6 mg/L.	[99]
	Immunomodulatory	No significant difference was found between conventional and ultrasonic extracts, both significantly suppressed the level of TNF-α production and LPS-activated macrophages. The measurement of IL-1β levels in intact macrophages showed that the ultrasonic extract caused a significant increase in IL-1β level compared to the control and conventional extract.	[32]
		Cleropane diterpenoids isolated from aerial part possessed a terminal *α*,*β*-unsaturated-*γ*-lactone moiety, and were assayed for their immunosuppressive activity via inhibiting the secretion of cytokines TNF-*α* and IL-6 in macrophages RAW264.7. Among them, (5*R*,8*R*,9*S*,10*R*)-18-*nor*-cleroda-2,13-dien-16,15-olide-4-one obviously suppressed the secretion of TNF-*α* and IL-6 with IC_50_ values of 8.55 and 13.65 μM, respectively.	[100]
	Anti-leishmanial	Taxodione isolated from root extract had significant anti-leishmanial activity with an IC_50_ value of 1.46 μM (0.46 mg/L) against *Leishmania donovani.*	[99]
	Antibacterial	Phenolic abietane diterpene isolated from root extract called ferruginol showed the strongest activity against *Streptococcus iniae*.	[99]
	Antifungal	Taxodione isolated from the root extract had IC_50_ values of 3.0 μM (0.93 mg/L) and 8.5 μM (2.67 mg/L) against *C. neoformans* and *Candida glabrata*, respectively.	[99]
	Antimicrobial	Taxodinone and ferruginol isolated from the root extract showed antibacterial activity against *S. aureus* and MRSA with IC_50_ values from 8.8 to 14.0 μM (2.78 to 4.00 mg/L) and from 8.4 to 9.5 μM (2.63 to 2.71 mg/L), respectively.	[99]
*S. trautvetteri*	No data available.		

AchE—acetylcholinesterase; BChE—butyrylcholinesterase; AGEs—advanced glycation end products; MPO—myeloperoxidase; ZOI—zone of inhibition; MIC—minimum inhibitory concentration; MBC—minimum bactericidal activity; LPO—lipid peroxidation; MAO-A—monoamine oxidase A; BHT—butylated hydroxytoluene; MMC—mitomycin C; CBPI—cytokinesis block proliferation index; MN—micronuclei; MDA-MB-231—human breast cancer cell line; HCT 116—human colorectal carcinoma cell line; MTT—3-(4,5-dimethylthiazol-2-yl)-2,5-diphenyl tetrazolium bromide; HDFn—neonatal human dermal fibroblasts.

## Data Availability

No new data were created or analyzed in this study.

## Abbreviations

ABTS	2,2′-azino-bis(3-ethylbenzothiazoline-6-sulfonic acid)
AChE	acetylcholinesterase
ADT	arogenate dehydratase
BAS	biologically active substances
BChE	butyrylcholinesterase
BHT	butylated hydroxytoluene
CUPRAC	cupric ion reducing antioxidant capacity
CBPI	cytokinesis block proliferation index
COX	cyclooxygenase
DDPH	(1-(2, 6-dimethylphenoxy)-2-(3, 4-dimethoxyphenylethylamino) propane hydrochloride)
EO	essential oil
FRAP	ferric reducing antioxidant power
GA	gallic acid
GC–MS	gas chromatography–mass spectrometry
HPLC–DAD–ESI–MS	high-performance liquid chromatography coupled with diode array detection and electrospray ionization mass spectrometry
HPLC–PDA	high-performance liquid chromatography coupled with photo diode array
HPTLC	high-performance thin-layer chromatography
QTOF–MS	quadrupole time-of-flight mass spectrometry
LPO	lipid peroxidation
LPS	lipopolysaccharide
LT	luteolin
MAE	microwave-assisted extraction
MAO-A	monoamine oxidase A
MBC	minimum bactericidal concentration
MDA	3,4-methylenedioxyamphetamine
MIC	minimum inhibitory concentration
MMC	mitomycin C
MPO	myeloperoxidase
MTT	3-[4,5-dimethylthiazol-2-yl]-2,5 diphenyl tetrazolium bromide)
NADES	natural deep eutectic solvent
QE	quercetin
RA	rosmarinic acid
RU	rutin
SFE	supercritical fluid extraction
TFC	total flavonoid content
TNF	tumor necrosis factor
TPC	total phenolic content
TRP	tryptophan
UPLC–Q/TOF	ultra-high performance liquid chromatography with quadrupole time-of-flight
VOC	volatile organic compound
ZOI	zone of inhibition
